# Statistical Inference of Truncated Normal Distribution Based on the Generalized Progressive Hybrid Censoring

**DOI:** 10.3390/e23020186

**Published:** 2021-02-02

**Authors:** Xinyi Zeng, Wenhao Gui

**Affiliations:** Department of Mathematics, Beijing Jiaotong University, Beijing 100044, China; 18271262@bjtu.edu.cn

**Keywords:** truncated normal distribution, generalized progressive hybrid censoring scheme, expectation maximization algorithm, Bayesian estimate, Tierney and Kadane approximation, importance sampling

## Abstract

In this paper, the parameter estimation problem of a truncated normal distribution is discussed based on the generalized progressive hybrid censored data. The desired maximum likelihood estimates of unknown quantities are firstly derived through the Newton–Raphson algorithm and the expectation maximization algorithm. Based on the asymptotic normality of the maximum likelihood estimators, we develop the asymptotic confidence intervals. The percentile bootstrap method is also employed in the case of the small sample size. Further, the Bayes estimates are evaluated under various loss functions like squared error, general entropy, and linex loss functions. Tierney and Kadane approximation, as well as the importance sampling approach, is applied to obtain the Bayesian estimates under proper prior distributions. The associated Bayesian credible intervals are constructed in the meantime. Extensive numerical simulations are implemented to compare the performance of different estimation methods. Finally, an authentic example is analyzed to illustrate the inference approaches.

## 1. Introduction

### 1.1. Truncated Normal Distribution

Normal distribution has played a crucial role in a diversity of research fields like reliability analysis and economics, as well as many other scientific developments. However, in many practical situations, experimental data are available from a certain range, so the truncated form of normal distribution is more applicable in actual life.

The truncated normal distribution, with its characteristic of practical, has gained some attention among researchers and interesting results have been obtained. Ref. [1] studied the maximum likelihood estimations for singly and doubly truncated normal distributions. Ref. [2] applied the approach of moments to estimate unknown parameters of singly truncated normal distributions from the first three sample moments. Ref. [3] investigated the estimators of unknown parameters from the normal distribution under the singly censored data. Ref. [4] utilized an iterative procedure to transform singly right censored samples into pseudo-complete samples and then estimated the parameters of interest through the transformed data. One can refer to [5] to find more details about the truncated normal distribution. Ref. [6] adopted the maximum likelihood estimation and Bayesian methods to estimate the unknown parameters for the truncated normal distribution under the progressive type-II censoring scheme. Meanwhile, optimal censoring plans under different optimality criteria had been discussed. While the above-mentioned works are based on the known truncation point. Ref. [7] developed a standard truncated normal distribution. The truncated mean and variance of this distribution are zero and one, respectively, regardless of the location of the truncation points. Ref. [8] considered the maximum likelihood estimators of unknown parameters with the known truncation point and the unknown truncation point respectively.

Generally, lifetime data are non-negative and under this circumstance, the left-truncated normal distribution (the truncation point is zero) can be applied to investigate statistical inference of unknown parameters.

Provided that a variable *X* is subject to a left-truncated normal distribution TN(μ,τ), which has a probability density function (pdf) when truncation ranges from zero to infinity.

$$f\left(x;\mu ,\tau \right)=\frac{e^{-\frac{1}{2\tau }{\left(x-\mu \right)}^{2}}}{\sqrt{2\pi \tau }\Phi \left(\frac{\mu }{\sqrt{\tau }}\right)},\;x>0,$$

where μ > 0 and τ > 0, μ is the mean of the corresponding normal distribution and τ is the variance accordingly. Φ(.) is the cumulative distribution function of the standard normal distribution.

The corresponding cumulative distribution function (cdf) takes the form as

$$F\left(x;\mu ,\tau \right)=1-\frac{1-\Phi (\frac{x-\mu }{\sqrt{\tau }})}{\Phi (\frac{\mu }{\sqrt{\tau }})},\;x>0.$$

The hazard rate function (hrf) of the left-truncated normal distribution at zero is expressed as below. In addition, we obtain the associated numeral characteristics such as the expectation and variance respectively.

$$h\left(x;\mu ,\tau \right)=\frac{e^{-\frac{1}{2\tau }{\left(x-\mu \right)}^{2}}}{\sqrt{2\pi \tau }\left(1-\Phi \left(\frac{x-\mu }{\sqrt{\tau }}\right)\right)},\;x>0.$$

$$E\left(X\right)=\mu -\frac{\sqrt{\tau }\phi \left(\frac{\mu }{\sqrt{\tau }}\right)}{\Phi (\frac{\mu }{\sqrt{\tau }})}$$

$$Var\left(X\right)=\tau \left[1+\frac{\frac{\mu }{\sqrt{\tau }}\phi \left(\frac{\mu }{\sqrt{\tau }}\right)}{\Phi (\frac{\mu }{\sqrt{\tau }})}-{\left(\frac{\phi (\frac{\mu }{\sqrt{\tau }})}{\Phi (\frac{\mu }{\sqrt{\tau }})}\right)}^{2}\right]$$

**Figure 1:**
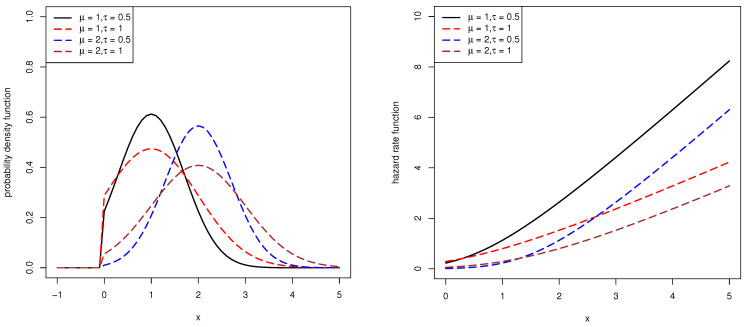
The pdfs and hrfs of the left-truncated normal distributions at zero.

Figure 1 presents the pdfs and hrfs of the left-truncated normal distributions when the truncation point is zero with different μ and τ. It is observed that the pdf is unimodal and the hrf is monotone increasing.

### 1.2. Generalized Progressive Hybrid Censoring Scheme

As technology has developed by leaps and bounds in the past few years, products are more reliable so that we can not get enough lifetime data to estimate the unknown parameters under the constraints of time and cost. As it is unavoidable to lose some experimental units during an experiment, an increasing number of researchers turned their attention to censored data. The earliest censoring schemes proposed are type-I and type-II censoring. Furthermore, combining type-I with type-II censoring schemes, researchers developed the hybrid censoring scheme. The features of all these schemes do not enable experimenters to withdraw any experimental unit at any stage before the experiment is over. Ref. [9] introduced the progressive censoring scheme afterwards. However, this scheme takes a huge amount of hours spent in conducting the experiment. In face of such defect, Ref. [10] developed an effective method-the progressive hybrid censoring scheme.

A progressive hybrid censoring sample can be generated as follows. First of all, *n* identical units are put into a test. We denote $X_{1},X_{2},\cdots ,X_{m}$ as the ordered failure times and $R=(R_{1},R_{2},\cdots ,R_{m})$ as the censoring scheme which satisfies that $\sum_{i=1}^{m}R_{i}+m=n$. Once the *m*-th failed unit happens or the threshold time *T* is reached, the test is stopped. That is, the stop time $T^{*}=\min\left\{X_{m},T\right\}$. When the first failure happens, we randomly remove $R_{1}$ units out of the test. On the occasion of the second failure, we withdraw $R_{2}$ survival units randomly from the test. Analogously, $R_{i}$ units are removed at random when the *i*-th failure occurs. Finally, either when the *m*-th failure has happened or the threshold time *T* has been reached, the rest of the survival units are taken out of the test together.

But in the progressive hybrid censoring scheme, we cannot get accurate estimates of unknown parameters since there might be only few failures before the threshold time *T* which is pre-fixed. For this reason, Ref. [11] introduced a generalized progressive hybrid censoring scheme (GPHCS). This censoring scheme can realize a compromise between time restriction and the number of failed observations by terminating the test at the random time $T_{end}=\max\left\{X_{k},T^{*}\right\}$. It guarantees that at least *k* failures can be observed before the terminal time $T_{end}$. Assume that a test starts with *n* identical units and the acceptable minimum number *k* of failures and the expected number *m* of failures observed are pre-fixed between zero and *n*, we choose the threshold time *T* and the censoring scheme that satisfies $\sum_{i=1}^{m}R_{i}+m=n$ ahead of the test. Similarly, we remove $R_{1}$ units randomly on the occasion of the first failure. On the arrival of the second failure, we randomly withdraw $R_{2}$ units from the rest of the experimental units. The procedure keeps repeating until the end time $T_{end}=\max\left\{X_{k},\min\left\{X_{m},T\right\}\right\}$ is reached.

**Figure 2:**
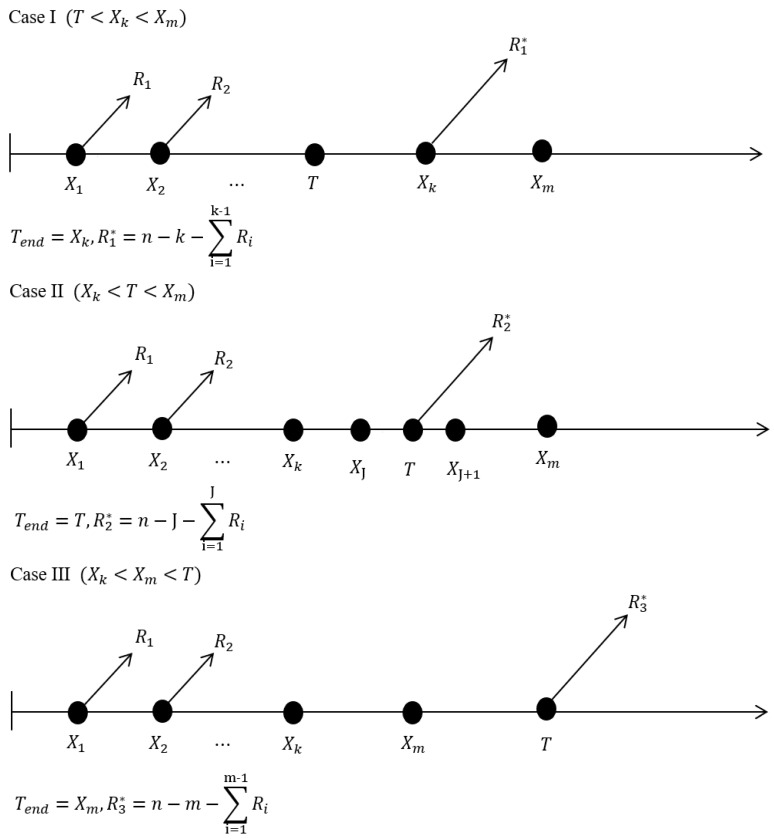
Three cases of the generalized progressive hybrid censoring scheme.

Schematically, Figure 2 gives the process that how to generate the generalized progressive hybrid censored data under different conditions of the pre-fixed time.

The generalized progressive hybrid censoring scheme modifies the terminal time $T_{end}$ to attain sufficient failed observations within a reasonable experimental period and brings about more accurate estimations of unknown parameters.

Since its higher efficiency of statistical inference, attention to generalized progressive hybrid censoring scheme has mounted. Ref. [11] employed the classical and Bayesian estimation techniques to estimate the entropy of Weibull distribution. Base on the method he introduced previously, Ref. [12] further derived the exact confidence intervals. Ref. [13] chose a competing risks model when data were sampled in the generalized progressive hybrid censoring scheme from an exponential distribution and derived the estimates through the maximum likelihood estimation approach and importance sampling method. On the basis of GPHCS, Ref. [14] investigated the two-parameter Rayleigh competing risk data adopting the maximum likelihood estimation and Gibbs sampling technique was employed to approximate the associated Bayes estimates.

The aim of our work is to obtain the classical and Bayesian estimations of the left-truncated normal distribution at zero when data are derived from the generalized progressive hybrid censoring. To begin with, the Newton–Raphson (N-R) algorithm is proposed to compute the maximum likelihood estimates (MLEs) of unknown parameters of TN(μ,τ). Another iterative approach—expectation maximization (EM) algorithm is also introduced to calculate the estimates. Subsequently, the observed Fisher information matrix and percentile bootstrap (Boot-p) method are considered to obtain the confidence interval estimations. In the Bayesian framework, we employ Tierney and Kadane (T-K) approximation and importance sampling (IS) technique to estimate Bayesian estimators. The associated higher posterior density (HPD) intervals are developed as well. Statistical inference of the left-truncated normal distribution at zero with the generalized progressive hybrid censored samples has not yet been carried out previously in terms of what we know about it.

The rest of this paper is made up of the following sections. The maximum likelihood estimators of μ and τ are theoretically derived respectively via the N-R approach and EM method in Section 2. In Section 3, we obtain the asymptotic confidence intervals using the asymptotic distributions of the MLEs, the asymptotic distributions of log-transformed MLEs and the Boot-p method. In Section 4, Bayes estimates of all unknown quantities are achieved applying T-K approximation under different loss functions. Besides, we figure out the Bayesian estimates of parameters using the importance sampling procedure. Based on this approach, the corresponding HPD intervals are developed. Numerical simulations and an analysis of one authentic example are carried out in Section 5. Finally, we arrive at some conclusive remarks in Section 6.

## 2. Maximum Likelihood Estimation

Our interest in this section is to obtain the maximum likelihood estimates of μ and τ with generalized progressive hybrid censored data. Based on the pdf and cdf of the truncated normal distribution when the left truncation point is zero, the likelihood and log-likelihood functions of three cases are expressed as

- Case I:
  $$\begin{array}{cc} L_{1} & \propto {\left(2\pi \tau \right)}^{-\frac{k}{2}}{\left[\Phi \left(\frac{\mu }{\sqrt{\tau }}\right)\right]}^{-n}e^{-\frac{1}{2\tau }\sum_{i=1}^{k}{\left(x_{i}-\mu \right)}^{2}}\prod_{i=1}^{k}{\left[1-\Phi \left(\frac{x_{i}-\mu }{\sqrt{\tau }}\right)\right]}^{R_{i}}\\ l_{1} & \propto -\frac{k}{2}\log\left(\tau \right)-n\log\Phi (\frac{\mu }{\sqrt{\tau }})-\frac{1}{2\tau }\sum_{i=1}^{k}{\left(x_{i}-\mu \right)}^{2}-\sum_{i=1}^{k}R_{i}\log\left(1-\Phi \left(\frac{x_{i}-\mu }{\sqrt{\tau }}\right)\right) \end{array}$$
- Case II:
  $$\begin{array}{cc} L_{2} & \propto {\left(2\pi \tau \right)}^{-\frac{D}{2}}{\left[\Phi \left(\frac{\mu }{\sqrt{\tau }}\right)\right]}^{-n}e^{-\frac{1}{2\tau }\sum_{i=1}^{D}{\left(x_{i}-\mu \right)}^{2}}\prod_{i=1}^{D}{\left[1-\Phi \left(\frac{x_{i}-\mu }{\sqrt{\tau }}\right)\right]}^{R_{i}}{\left[1-\Phi \left(\frac{T-\mu }{\sqrt{\tau }}\right)\right]}^{R_{2}^{*}}\\ l_{2} & \propto -\frac{D}{2}\log\left(\tau \right)-n\log\Phi (\frac{\mu }{\sqrt{\tau }})-\frac{1}{2\tau }\sum_{i=1}^{D}{\left(x_{i}-\mu \right)}^{2}-\sum_{i=1}^{D}R_{i}\log\left(1-\Phi \left(\frac{x_{i}-\mu }{\sqrt{\tau }}\right)\right)\\ \; & +R_{2}^{*}\log\left(1-\Phi \left(\frac{T-\mu }{\sqrt{\tau }}\right)\right) \end{array}$$
- Case III:
  $$\begin{array}{cc} L_{3} & \propto {\left(2\pi \tau \right)}^{-\frac{m}{2}}{\left[\Phi \left(\frac{\mu }{\sqrt{\tau }}\right)\right]}^{-n}e^{-\frac{1}{2\tau }\sum_{i=1}^{m}{\left(x_{i}-\mu \right)}^{2}}\prod_{i=1}^{m}{\left[1-\Phi \left(\frac{x_{i}-\mu }{\sqrt{\tau }}\right)\right]}^{R_{i}}\\ l_{3} & \propto -\frac{m}{2}\log\left(\tau \right)-n\log\Phi (\frac{\mu }{\sqrt{\tau }})-\frac{1}{2\tau }\sum_{i=1}^{m}{\left(x_{i}-\mu \right)}^{2}-\sum_{i=1}^{m}R_{i}\log\left(1-\Phi \left(\frac{x_{i}-\mu }{\sqrt{\tau }}\right)\right) \end{array}$$

Then the likelihood and log-likelihood functions for three kinds of cases can be combined in a general expression as

$$L\left(\mu ,\tau |\tilde{x}\right)\propto \tau^{-\frac{J}{2}}e^{-\frac{1}{2\tau }\sum_{i=1}^{J}{\left(x_{i}-\mu \right)}^{2}}{\left[\Phi \left(\frac{\mu }{\sqrt{\tau }}\right)\right]}^{-n}\prod_{i=1}^{J}{\left[1-\Phi \left(\frac{x_{i}-\mu }{\sqrt{\tau }}\right)\right]}^{R_{i}}H\left(\mu ,\tau \right)$$

(1) $$\begin{array}{cc} l(\mu ,\tau |\tilde{x})=l\propto & -\frac{J}{2}\log\left(\tau \right)-n\log\Phi \left(\frac{\mu }{\sqrt{\tau }}\right)-\frac{1}{2\tau }\sum_{i=1}^{J}{\left(x_{i}-\mu \right)}^{2}\\ \; & +\sum_{i=1}^{J}R_{i}\log\left(1-\Phi \left(\frac{x_{i}-\mu }{\sqrt{\tau }}\right)\right)+h\left(\mu ,\tau \right). \end{array}$$

where $\tilde{x}=\left(x_{1},x_{2},\cdots ,x_{J}\right)$ denotes the observed data and J=k, $R_{k}=R_{1}^{*}$, H(μ,τ)=1, h(μ,τ)=0 for Case I; J=D,$H\left(\mu ,\tau \right)={\left[1-\Phi \left(\frac{T-\mu }{\sqrt{\tau }}\right)\right]}^{R_{2}^{*}}$, $h\left(\mu ,\tau \right)=R_{2}^{*}\log\left(1-\Phi \left(\frac{T-\mu }{\sqrt{\tau }}\right)\right)$ for Case II; J=m, $R_{m}=R_{3}^{*}$, H(μ,τ)=1, h(μ,τ)=0 for Case III.

Next, take the first derivatives of (1) for μ and τ respectively and make them equal to zero. A set of score equations can be attained as follows.

$$\frac{\partial l}{\partial \mu }=\frac{1}{\sqrt{\tau }}\sum_{i=1}^{J}\eta_{i}+\frac{1}{\sqrt{\tau }}\sum_{i=1}^{J}\frac{R_{i}\phi \left(\eta_{i}\right)}{1-\Phi (\eta_{i})}-\frac{n}{\sqrt{\tau }}\frac{\phi (\eta )}{\Phi (\eta )}+h_{1}\left(\mu ,\tau \right)=0$$

$$\frac{\partial l}{\partial \tau }=-\frac{J}{2\tau }+\frac{1}{2\tau }\sum_{i=1}^{J}\eta_{i}^{2}+\frac{1}{2\tau }\sum_{i=1}^{J}\frac{R_{i}\eta_{i}\phi \left(\eta_{i}\right)}{1-\Phi (\eta_{i})}+\frac{n\eta }{2\tau }\frac{\phi (\eta )}{\Phi (\eta )}+h_{2}\left(\mu ,\tau \right)=0$$

where $\eta =\frac{\mu }{\sqrt{\tau }}$, $\eta_{i}=\frac{x_{i}-\mu }{\sqrt{\tau }}$, $\eta_{T}=\frac{T-\mu }{\sqrt{\tau }}$ and $H_{1}\left(\mu ,\tau \right)=0$, $H_{2}\left(\mu ,\tau \right)=0$ for Case I and Case II; $h_{1}\left(\mu ,\tau \right)=\frac{R_{2}^{*}}{\sqrt{\tau }}\frac{\phi (\eta_{T})}{1-\Phi (\eta_{T})}$, $h_{2}\left(\mu ,\tau \right)=\frac{R_{2}^{*}\eta_{T}}{2\tau^{2}}\frac{\phi (\eta_{T})}{1-\Phi (\eta_{T})}$ for Case III.

The maximum likelihood estimates of unknown parameters are the solutions to the above equations. Apparently, the expressions of μ and τ are involved in a nonlinear problem and the analytic solutions are not available. Therefore, we have to depend on some numerical methods such as the N-R method and EM algorithm to approximate the values of unknown parameters.

### 2.1. Newton–Raphson Algorithm

Since the first and second-order derivations of the log-likelihood function are available, the Newton–Raphson algorithm is appropriate to maximize the log-likelihood function.

The second-order derivations of (1) for the parameters are given by

$$\begin{array}{cc} l_{20}=\frac{\partial^{2}l}{\partial \mu^{2}}\; & =-\frac{J}{\tau }-\frac{n}{\tau }\left(Q^{\prime }-Q^{2}\right)-\sum_{i=1}^{J}\frac{R_{i}}{\tau }\left(Q_{i}^{\prime }-Q_{i}^{2}\right)+h_{20}\left(\mu ,\tau \right) \end{array}$$

$$\begin{array}{cc} l_{11} & =\frac{\partial^{2}l}{\partial \mu \partial \tau }=-\frac{1}{\tau^{3/2}}\sum_{i=1}^{J}\eta_{i}+\frac{n}{2\tau^{3/2}}Q+\frac{n\mu }{2\tau^{2}}\left(Q^{\prime }-Q^{2}\right)-\sum_{i=1}^{J}\frac{R_{i}}{2\tau^{3/2}}Q_{i}\\ \; & -\sum_{i=1}^{J}\frac{R_{i}\eta_{i}}{2\tau^{3/2}}\left(Q_{i}^{\prime }-Q_{i}^{2}\right)+h_{11}\left(\mu ,\tau \right) \end{array}$$

$$\begin{array}{cc} l_{02} & =\frac{\partial^{2}l}{\partial \tau^{2}}=\frac{J}{2\tau^{2}}-\frac{1}{\tau^{2}}\sum_{i=1}^{J}\eta_{i}^{2}-\frac{3n\mu }{4\tau^{5/2}}Q-\frac{n\mu^{2}}{4\tau^{3}}\left(Q^{\prime }-Q^{2}\right)-\sum_{i=1}^{J}\frac{3R_{i}\eta_{i}}{4\tau^{2}}Q_{i}\\ \; & -\sum_{i=1}^{J}\frac{R_{i}\eta_{i}^{2}}{4\tau^{2}}\left(Q_{i}^{\prime }-Q_{i}^{2}\right)+h_{02}\left(\mu ,\tau \right) \end{array}$$

with

$$\begin{array}{cc} h_{20}\left(\mu ,\tau \right) & =-\frac{R_{2}^{*}}{\tau }\left(Q_{T}^{\prime }+Q_{T}^{2}\right)\\ h_{11}\left(\mu ,\tau \right) & =-\frac{R_{2}^{*}}{2\tau^{3/2}}\left(Q_{T}+\eta_{T}Q_{T}^{\prime }+\eta_{T}Q_{T}^{2}\right)\\ h_{22}\left(\mu ,\tau \right) & =-\frac{R_{2}^{*}}{4\tau^{2}}\left(3\eta_{T}Q_{T}+\eta_{T}^{2}Q_{T}^{\prime }+\eta_{T}^{2}Q_{T}^{2}\right) \end{array}$$

where $\eta =\mu /\sqrt{\tau }$, $Q^{\prime }=\phi^{\prime }\left(\eta \right)/\Phi \left(\eta \right)$, Q=ϕ(η)/Φ(η), $\eta_{i}=\left(x_{i}-\mu \right)/\sqrt{\tau }$, $Q_{i}^{\prime }=\phi^{\prime }\left(\eta_{i}\right)/\left(1-\Phi \left(\eta_{i}\right)\right)$ and $Q_{i}=\phi \left(\eta_{i}\right)/\left(1-\Phi \left(\eta_{i}\right)\right)$. Similarly, $\eta_{T}=\left(T-\mu \right)/\sqrt{\tau }$, $Q_{T}^{\prime }=\phi^{\prime }\left(\eta_{T}\right)/\left(1-\Phi \left(\eta_{T}\right)\right)$ and $Q_{T}=\phi \left(\eta_{T}\right)/\left(1-\Phi \left(\eta_{T}\right)\right)$.

The estimates can be updated by

$$\left[\begin{array}{c} \mu_{\left(l+1\right)}\\ \tau_{\left(l+1\right)} \end{array}\right]=\left[\begin{array}{c} \mu_{\left(l\right)}\\ \tau_{\left(l\right)} \end{array}\right]-J{\left(\mu_{\left(l\right)},\tau_{\left(l\right)}\right)}^{-1}\left[\begin{array}{c} \mu_{\left(l\right)}\\ \tau_{\left(l\right)} \end{array}\right]$$

where

$$J\left(\mu_{\left(l\right)},\tau_{\left(l\right)}\right)={\left[\begin{array}{cc} l_{20} & l_{11}\\ l_{11} & l_{02} \end{array}\right]}_{\left(\mu ,\tau \right)=\left(\mu_{\left(l\right)},\tau_{\left(l\right)}\right)}$$

The process repeats until $|\mu_{l+1}-\mu_{l}|<\varepsilon$ and $|\tau_{l+1}-\tau_{l}|<\varepsilon$, where ε is pre-fixed as the tolerance limit.

### 2.2. Expectation Maximization Algorithm

Here EM algorithm discussed in [15], is employed to obtain the maximum likelihood estimates of μ and τ for the left-truncated normal distribution. It is an effective procedure to calculate the MLEs in the case of censored data. The expectation step (E-step) and maximization step (M-step) are two steps in the progress of the EM algorithm to calculate estimates for the given model. The former is to compute adequate information of censored data based on observed data, whereas the latter one is to re-estimate current parameters.

As mentioned earlier, we can only observe *J* failure units for three cases under consideration. Assume that $X=(X_{1},X_{2},\cdots ,X_{J})$ stands for the observed data which is subject to the left-truncated normal distribution at zero and $Z=\left(Z_{1},Z_{2},\cdots ,Z_{J}\right)⋃\left(Z_{T_{j}},j=1,2,\cdots ,R_{T}\right)$ denotes censored data, where $Z_{ij}$, i=1, 2,⋯, *J*, j=1, 2, ⋯, $R_{J}$ is the *j*-th unit that was withdrawn at the failure time $X_{i}$ and $Z_{T_{j}}$, j=1, 2, ⋯, $R_{T}$, is the *j*-th unit that was withdrawn at the end time $T_{end}$ in Case II. Thus, we denote the complete data as C=(X,Z), then the likelihood function of complete data under the GPHCS takes the form as

$$\begin{array}{cc} L_{c}\left(\mu ,\tau \right) & =\prod_{i=1}^{J}\left[f\left(x_{i}\right)\prod_{j=1}^{R_{i}}f\left(z_{ij}\right)\right]\prod_{j=1}^{R_{T}}f\left(z_{Tj}\right)\\ = & {\left(2\pi \tau \right)}^{-\frac{n}{2}}{\left(\Phi \left(\frac{\mu }{\sqrt{\tau }}\right)\right)}^{-n}e^{-\frac{1}{2\tau }\left[\sum_{i=1}^{J}{\left(x_{i}-\mu \right)}^{2}+\sum_{i=1}^{J}\sum_{j=1}^{R_{i}}{\left(z_{ij}-\mu \right)}^{2}+\sum_{j=1}^{R_{T}}{\left(z_{Tj}-\mu \right)}^{2}\right]}. \end{array}$$

Leaving out the constant term, the corresponding log-likelihood function is transformed into

$$\begin{array}{cc} l_{c}\left(\mu ,\tau \right) & =-\frac{n}{2}\log\left(\tau \right)-n\log\Phi (\eta )-\frac{n\mu^{2}}{2\tau }-\frac{1}{2\tau }\left(\sum_{i=1}^{J}x_{i}^{2}+\sum_{i=1}^{J}\sum_{j=1}^{R_{i}}z_{ij}^{2}+\sum_{j=1}^{R_{T}}z_{Tj}^{2}\right)\\ \; & +\frac{\mu }{\tau }\left(\sum_{i=1}^{J}x_{i}+\sum_{i=1}^{J}\sum_{j=1}^{R_{i}}z_{ij}+\sum_{j=1}^{R_{T}}z_{Tj}\right). \end{array}$$

Following two steps of the EM algorithm implemented in [16], we can get the ‘pseudo-log-likelihood’ function by utilizing the associated expected values to take place of the censored data.

E-step: Under the complete data, the ‘pseudo-log-likelihood’ function is

(2) $$\begin{array}{cc} l_{s}\left(\mu ,\tau \right)= & -\frac{n}{2}\log\left(\tau \right)-n\log\Phi (\eta )-\frac{n\mu^{2}}{2\tau }+\frac{\mu }{\tau }\left(\sum_{i=1}^{J}x_{i}+\sum_{i=1}^{J}\sum_{j=1}^{R_{i}}E\left(z_{ij}|z_{ij}>x_{i}\right)+\sum_{j=1}^{R_{T}^{*}}E\left(z_{Tj}|z_{Tj}>T\right)\right)\\ \; & -\frac{1}{2\tau }\left(\sum_{i=1}^{J}x_{i}^{2}+\sum_{i=1}^{J}\sum_{j=1}^{R_{i}}E\left(z_{ij}^{2}|z_{ij}>x_{i}\right)+\sum_{j=1}^{R_{T}}E\left(z_{Tj}^{2}|z_{Tj}>T\right)\right). \end{array}$$

For i=1, 2,⋯, *J*; j=1, 2,⋯, $R_{J}$, the conditional expectations mentioned above are deduced as

(3) $$\begin{array}{cc} E(z_{ij}|z_{ij}>x_{i}) & =\mu +\sqrt{\tau }Q_{i}=A\left(x_{i},\mu_{\left(l\right)},\tau_{\left(l\right)}\right)\\ E(z_{ij}^{2}|z_{ij}>x_{i}) & =\mu^{2}+\tau +2\mu \sqrt{\tau }Q_{i}+\tau \eta_{i}Q_{i}=B\left(x_{i},\mu_{\left(l\right)},\tau_{\left(l\right)}\right), \end{array}$$

By analogy, for j=1, 2, ⋯, $R_{T}$,

(4) $$\begin{array}{cc} E(z_{Tj}|z_{Tj}>T) & =\mu +\sqrt{\tau }Q_{T}=C\left(T,\mu_{\left(l\right)},\tau_{\left(l\right)}\right)\\ E(z_{Tj}^{2}|z_{Tj}>T) & =\mu^{2}+\tau +2\mu \sqrt{\tau }Q_{T}+\tau \eta_{T}Q_{T}=D\left(T,\mu_{\left(l\right)},\tau_{\left(l\right)}\right). \end{array}$$

where $\eta =\mu /\sqrt{\tau }$, $\eta_{i}=\left(x_{i}-\mu \right)/\sqrt{\tau }$, $\eta_{T}=\left(T-\mu \right)/\sqrt{\tau }$, $Q_{i}=\phi \left(\eta_{i}\right)/\left(1-\Phi \left(\eta_{i}\right)\right)$ and $Q_{T}=\left(T-\mu \right)/\sqrt{\tau }$.

By substituting (3) and (4) into (2), the ‘pseudo log-likelihood’ function becomes

(5) $$\begin{array}{cc} l_{s}\left(\mu ,\tau \right)= & -\frac{n}{2}\log\tau -n\log\Phi (\frac{\mu }{\sqrt{\tau }})-\frac{n\mu^{2}}{2\tau }+\frac{\mu }{\tau }\left(\sum_{i=1}^{J}x_{i}+\sum_{i=1}^{J}\sum_{j=1}^{R_{i}}A\left(x_{i},\mu_{\left(l\right)},\tau_{\left(l\right)}\right)+\sum_{j=1}^{R_{T}}C\left(T,\mu_{\left(l\right)},\tau_{\left(l\right)}\right)\right)\\ \; & -\frac{1}{2\tau }\left(\sum_{i=1}^{J}x_{i}^{2}+\sum_{i=1}^{J}\sum_{j=1}^{R_{i}}B\left(x_{i},\mu_{\left(l\right)},\tau_{\left(l\right)}\right)+\sum_{j=1}^{R_{T}}D\left(T,\mu_{\left(l\right)},\tau_{\left(l\right)}\right)\right). \end{array}$$

M-step: The major purpose of this step is to maximize the ‘pseudo log-likelihood’ function to calculate the next iterate. Let (5) take derivatives with regard to μ and τ respectively and equal them to zero, the corresponding score equations can be obtained as below. Suppose that $\left(\mu_{\left(l\right)},\tau_{\left(l\right)}\right)$ is the *l*-th iteration estimate of (μ,τ), $\left(\mu_{\left(l+1\right)},\tau_{\left(l+1\right)}\right)$ is derived afterwards.

$$n\mu_{\left(l+1\right)}+n\sqrt{\tau_{\left(l\right)}}\frac{\phi (\frac{\mu_{\left(l+1\right)}}{\sqrt{\tau_{\left(l\right)}}})}{\Phi (\frac{\mu_{\left(l+1\right)}}{\sqrt{\tau_{\left(l\right)}}})}-\left(\sum_{i=1}^{J}x_{i}+\sum_{i=1}^{J}\sum_{j=1}^{R_{i}}A\left(x_{i},\mu_{\left(l\right)},\tau_{\left(l\right)}\right)+\sum_{j=1}^{R_{T}}C\left(x_{i},\mu_{\left(l\right)},\tau_{\left(l\right)}\right)\right)=0$$

$$\begin{array}{cc} n\tau_{\left(l+1\right)}- & n\mu_{\left(l+1\right)}^{2}-n\mu_{\left(l+1\right)}\sqrt{\tau_{\left(l+1\right)}}\frac{\phi (\frac{\mu_{\left(l+1\right)}}{\sqrt{\tau_{\left(l+1\right)}}})}{\Phi (\frac{\mu_{\left(l+1\right)}}{\sqrt{\tau_{\left(l+1\right)}}})}+2\mu_{\left(l+1\right)}\left(\sum_{i=1}^{J}x_{i}+\sum_{i=1}^{J}\sum_{j=1}^{R_{i}}A\left(x_{i},\mu_{\left(l\right)},\tau_{\left(l\right)}\right)\right.\\ \; & \left.+\sum_{j=1}^{R_{T}}C\left(x_{i},\mu_{\left(l\right)},\tau_{\left(l\right)}\right)\right)-\left(\sum_{i=1}^{J}x_{i}^{2}+\sum_{i=1}^{J}\sum_{j=1}^{R_{i}}B\left(x_{i},\mu_{\left(l\right)},\tau_{\left(l\right)}\right)+\sum_{j=1}^{R_{T}}D\left(x_{i},\mu_{\left(l\right)},\tau_{\left(l\right)}\right)\right)=0 \end{array}$$

Next upgrade $\mu_{\left(l\right)}$ and $\tau_{\left(l\right)}$ into $\mu_{\left(l+1\right)}$ and $\tau_{\left(l+1\right)}$ by soving the equations shown above. The E-step and M-step continue until the precision satisfies the tolerance limit that is fixed ahead of time $\left|\mu_{\left(l+1\right)}-\mu_{\left(l\right)}\right|+\left|\tau_{\left(l+1\right)}-\tau_{\left(l\right)}\right|<\varepsilon$. By that time, the convergence values are seen as the estimated values of μ and τ based on EM algorithm.

## 3. Confidence Interval Estimation

In this section, confidence interval estimates are provided for unknown parameters μ and τ using the MLE-based asymptotic confidence intervals (ACIs), the log-transformed MLE-based asymptotic confidence intervals (Log-CIs) and bootstrap confidence intervals (Boot-p CIs).

### 3.1. Asymptotic Confidence Intervals for Mles

Based on the asymptotic normal property of the MLEs, the asymptotic distribution of $\left({\hat{\mu }}_{M},{\hat{\tau }}_{M}\right)$ is $\left({\hat{\mu }}_{M},{\hat{\tau }}_{M}\right)$→$N_{2}\left(\left(\mu ,\tau \right),I^{-1}\left(\mu ,\tau \right)\right)$, where ${\hat{\mu }}_{M}$, ${\hat{\tau }}_{M}$ are the MLEs of μ and τ, $I^{-1}\left(\mu ,\tau \right)$ stands for the inverse of the Fisher information matrix. Then the variance-covariance Fisher information matrix of (μ,τ) is able to obtain theoretically from the following of Fisher information matrix.

(6) $$I^{-1}\left(\mu ,\tau \right)={\left[E\left(-\frac{\partial^{2}l}{\partial \mu^{i}\partial \tau^{j}}\right)\right]}^{-1}\;i+j=2,i,j=0,1,2$$

At times it’s troublesome to figure out the exact Fisher information matrix. Therefore, $I_{obs}^{-1}\left({\hat{\mu }}_{M},{\hat{\tau }}_{M}\right)$ the inverse of the observed Fisher information matrix is employed to make an estimation of $I^{-1}\left(\mu ,\tau \right)$. Here we express the observed Fisher information matrix of unknown variables μ and τ as the following form.

(7) $$I_{obs}\left(\mu ,\tau \right)=-{\left(\begin{array}{ccc} \frac{\partial^{2}l}{\partial \mu^{2}} & \frac{\partial^{2}l}{\partial \mu \partial \tau }\\ \; & \frac{\partial^{2}l}{\partial \tau \partial \mu } & \frac{\partial^{2}l}{\partial \tau^{2}} \end{array}\right)}_{\left(\mu ,\tau \right)=\left({\hat{\mu }}_{M},{\hat{\tau }}_{M}\right)}.$$

The elements of the matrix (7) are calculated in Section 2.

Subsequently, the asymptotic variance-covariance matrix is derived from

(8) $$I_{obs}^{-1}\left(\mu ,\tau \right)=\left(\begin{array}{cc} Var({\hat{\mu }}_{M}) & Cov({\hat{\mu }}_{M},{\hat{\tau }}_{M})\\ Cov({\hat{\tau }}_{M},{\hat{\mu }}_{M}) & Var({\hat{\tau }}_{M}) \end{array}\right)=\left(\begin{array}{ccc} \; & v_{11} & v_{12}\\ \; & v_{21} & v_{22} \end{array}\right).$$

Based on the matrix (8), the approximate variance of two parameters μ and τ can be derived, then we can obtain the 100$\left(1-\zeta \right)\%$ ACIs for these two parameters

$$\left({\hat{\mu }}_{M}-Z_{1-\zeta /2}\sqrt{v_{11}},{\hat{\mu }}_{M}+Z_{1-\zeta /2}\sqrt{v_{11}}\right),\;\left({\hat{\tau }}_{M}-Z_{1-\zeta /2}\sqrt{v_{22}},{\hat{\tau }}_{M}+Z_{1-\zeta /2}\sqrt{v_{22}}\right).$$

where $Z_{1-\zeta /2}$ satisfies $P\left(X\leq Z_{1-\zeta /2}\right)=1-\frac{\zeta }{2}$ when *X* follows the standard normal distribution.

### 3.2. Asymptotic Confidence Intervals for Log-Transformed Mles

Occasionally, the lower bound of ACI is less than zero. To conquer this setback, the logarithmic transformation and delta method is suggested to make sure that the lower bound of ACI is nonnegative.

Let α=(μ,τ) denotes the unknown parameter vector, in accordance to [17], the distribution of

$$\log\left(\hat{\alpha }\right)=\frac{\log\left(\hat{\alpha }\right)-\log\left(\alpha \right)}{\sqrt{Var(\log\left(\hat{\alpha }\right))}}$$

is approximately subject to the standard normal distribution, where $\alpha_{1}$ = μ and $\alpha_{2}$ = τ. Hence, a 100$\left(1-\zeta \right)\%$ logarithmic transformation confidence interval for $\alpha_{i}$ is further constructed as

$$\left(\frac{\hat{\alpha_{i}}}{\exp\left(Z_{1-\zeta /2}\sqrt{Var(\log\hat{\alpha_{i}})}\right)},\hat{\alpha_{i}}\exp\left(Z_{1-\zeta /2}\sqrt{Var(\log\hat{\alpha_{i}})}\right)\right),\;i=1,2$$

where $\hat{\alpha_{i}}$ is the MLE of $\alpha_{i}$ and $Var\left(\log\hat{\alpha_{i}}\right)=\frac{Var(\hat{\alpha_{i}})}{\alpha_{i}^{2}}=\frac{I^{-1}{\left(\hat{\alpha }\right)}_{ii}}{\alpha_{i}^{2}}$, i=1,2.

### 3.3. Percentile Bootstrap Approach

The methods of asymptotic confidence intervals introduced above are both originated from the laws of large numbers. They do not perform effectively when faced with a small sample size. We suggest the percentile bootstrap approach to overcome the drawback and construct Boot-p CIs for μ and τ as well. According to [18], the following steps can be implemented to generate the bootstrap samples to develop Boot-p CIs.

Step 1: Calculate the MLEs of two parameters μ and τ from the original generalized progressive hybrid censored sample.
Step 2: Utilize the same censoring scheme (T,n,m,k,R) and ${\hat{\mu }}_{M}$ and ${\hat{\tau }}_{M}$ to generate a generalized progressive hybrid censored bootstrap sample.
Step 3: Calculate the bootstrap estimators of μ and τ, denote as $\mu^{*}$ and $\tau^{*}$, from the bootstrap sample of truncated normal distribution.
Step 4: Perform Step 2 and Step 3, *N* times to obtain a sequence of bootstrap estimators.
Step 5: Sort $\mu^{1*},\mu^{2*},\cdots ,\mu^{N*}$ and $\tau^{1*},\tau^{2*},\cdots ,\tau^{N*}$ in ascending order respectively. Then we get $\mu_{\left(1\right)}^{*},\mu_{\left(2\right)}^{*},\cdots ,\mu_{\left(N\right)}^{*}$ and $\tau_{\left(1\right)}^{*},\tau_{\left(2\right)}^{*},\cdots ,\tau_{\left(N\right)}^{*}$.
Step 6: The 100$\left(1-\zeta \right)\%$ Boot-p CIs of μ and τ are $\left(\mu_{\left(N(\zeta /2)\right)}^{*},\mu_{\left(N(1-\zeta /2)\right)}^{*}\right)$ and $(\tau_{\left(N(\zeta /2)\right)}^{*},$$\tau_{\left(N(1-\zeta /2)\right)}^{*})$ respectively.

## 4. Bayes Estimation

Bayes point and interval estimations can be evaluated for unknown variables μ and τ belong to truncated normal distribution under the GPHCS in this section. All the Bayes estimations can be deduced theoretically under symmetric and asymmetric loss functions using Tierney and Kadane approximation and the importance sampling technique.

### 4.1. Prior and Posterior Distribution

Since a conjugate prior distribution for μ and τ does not exist, we make the same assumption as in [6] that μ and τ have a conditional bivariate prior distribution as the following form.

$$\pi \left(\mu ,\tau \right)=\pi_{1}\left(\tau \right)\pi_{2}\left(\mu |\tau \right)$$

where

$$\pi_{1}\left(\tau \right)=\frac{{\left(\frac{d}{2}\right)}^{c}}{\Gamma (c)}{\left(\frac{1}{\tau }\right)}^{c+1}e^{-\frac{d}{2\tau }}$$

$$\pi_{2}\left(\mu |\tau \right)=\frac{e^{-\frac{b}{2\tau }{\left(\mu -a\right)}^{2}}}{\sqrt{\frac{2\pi \tau }{b}}\Phi \left(a\frac{\sqrt{b}}{\sqrt{\tau }}\right)}$$

The prior distribution of τ is the inverse Gamma distribution IG(*c*, d/2). While under the circumstance that τ is known, μ follows the truncated normal distribution TN(*a*, τ/b) when the truncation point is zero. Here *a*, *b*, *c*, and *d* are treated as the hyper-parameters, whose domain range from zero to positive infinity.

Therefore the joint prior distribution is written as

(9) $$\pi_{0}\left(\mu ,\tau \right)\propto \frac{1}{\Phi \left(\frac{a\sqrt{b}}{\sqrt{\tau }}\right)}{\left(\frac{1}{\tau }\right)}^{c+\frac{3}{2}}e^{-\frac{1}{2\tau }\left[b{\left(\mu -a\right)}^{2}+d\right]}.$$

Using (1) and (9), the posterior distribution of the left-truncated normal distribution TN(μ,τ) at zero is

(10) $$\begin{array}{cc} \pi (\mu ,\tau |\tilde{x})=P & \frac{{\left[\Phi \left(\frac{\mu }{\sqrt{\tau }}\right)\right]}^{-n}}{\Phi \left(\frac{a\sqrt{b}}{\sqrt{\tau }}\right)}{\left(\frac{1}{\tau }\right)}^{(c+\frac{1+J}{2})+1}e^{-\frac{1}{2\tau }\left[d+\sum_{i=1}^{J}{\left(x_{i}-\mu \right)}^{2}+b{\left(\mu -a\right)}^{2}\right]}\\ \; & \times \prod_{i=1}^{J}{\left(1-\Phi \left(\frac{x_{i}-\mu }{\sqrt{\tau }}\right)\right)}^{R_{i}}H\left(\mu ,\tau \right). \end{array}$$

where *P* is the normalizing constant satisfying that

$$\begin{array}{cc} P^{-1}= & \int_{0}^{\infty }\int_{0}^{\infty }\frac{{\left[\Phi \left(\frac{\mu }{\sqrt{\tau }}\right)\right]}^{-n}}{\Phi \left(\frac{a\sqrt{b}}{\sqrt{\tau }}\right)}{\left(\frac{1}{\tau }\right)}^{c+\frac{3+J}{2}}e^{-\frac{1}{2\tau }\left[d+\sum_{i=1}^{J}{\left(x_{i}-\mu \right)}^{2}+b{\left(\mu -a\right)}^{2}\right]}\\ \; & \times \prod_{i=1}^{J}{\left(1-\Phi \left(\frac{x_{i}-\mu }{\sqrt{\tau }}\right)\right)}^{R_{i}}H\left(\mu ,\tau \right)d\mu d\tau . \end{array}$$

To start with, ℵ(μ,τ) denotes a function of μ and τ. So the expectation of ℵ(μ,τ) given $\tilde{x}$ is shown as

(11) $$E\left[ℵ\left(\mu ,\tau \right)|\tilde{x}\right]=\frac{\int_{0}^{\infty }\int_{0}^{\infty }ℵ\left(\mu ,\tau \right)L\left(\mu ,\tau |\tilde{x}\right)\pi_{0}\left(\mu ,\tau \right)d\mu \;d\tau }{\int_{0}^{\infty }\int_{0}^{\infty }L\left(\mu ,\tau |\tilde{x}\right)\pi_{0}\left(\mu ,\tau \right)d\mu \;d\tau }.$$

### 4.2. Loss Functions

In the Bayesian framework, the Bayes estimate of a function ℵ(μ,τ) can be derived based on a prescribed loss function. We discuss three kinds of loss functions, namely squared error, general entropy, and linex loss functions.

- Squared error loss function

Squared error loss (SEL) function is the most universally applicable loss function to obtain Bayes estimators of unknown parameters. We can express its definition as

$$L_{s}\left(\varpi ,\hat{\varpi }\right)={\left(\hat{\varpi }-\varpi \right)}^{2}.$$

In the following cases, we denote $\hat{\varpi }$ as an estimator of ϖ.

Under this circumstance, the corresponding Bayes estimator ${\hat{\varpi }}_{s}$ of ϖ can be derived from

$$\varpi_{s}=E\left(\varpi |\tilde{x}\right).$$

Then we can obtain the Bayes estimate $\hat{ℵ}{\left(\mu ,\tau \right)}_{s}$ of ℵ(μ,τ) under SEL function

(12) $$\hat{ℵ}{\left(\mu ,\tau \right)}_{s}=\frac{\int_{0}^{\infty }\int_{0}^{\infty }L\left(\mu ,\tau |\tilde{x}\right)ℵ\left(\mu ,\tau \right)\pi_{0}\left(\mu ,\tau \right)d\mu \;d\tau }{\int_{0}^{\infty }\int_{0}^{\infty }L\left(\mu ,\tau |\tilde{x}\right)\pi_{0}\left(\mu ,\tau \right)d\mu \;d\tau }.$$

- General entropy loss function

The expression of general entropy loss (GEL) function is

$$L_{e}\left(\varpi ,\hat{\varpi }\right)={\left(\frac{\hat{\varpi }}{\varpi }\right)}^{q}-qlog\left(\frac{\hat{\varpi }}{\varpi }\right)-1,\;q\neq 0.$$

Under this circumstance, the Bayes estimator ${\hat{\varpi }}_{e}$ of ϖ can be derived from

$$\varpi_{e}=E{\left(\varpi^{-q}|X\right)}^{\frac{1}{q}}.$$

Finally, the Bayes estimate $\hat{ℵ}{\left(\mu ,\tau \right)}_{e}$ of ℵ(μ,τ) results to be the following form based on GEL function.

(13) $$\hat{ℵ}{\left(\mu ,\tau \right)}_{e}={\left[\frac{\int_{0}^{\infty }\int_{0}^{\infty }ℵ{\left(\mu ,\tau \right)}^{-q}L\left(\mu ,\tau |\tilde{x}\right)\pi_{0}\left(\mu ,\tau \right)d\mu \;d\tau }{\int_{0}^{\infty }\int_{0}^{\infty }L\left(\mu ,\tau |\tilde{x}\right)\pi_{0}\left(\mu ,\tau \right)d\mu \;d\tau }\right]}^{-\frac{1}{q}}.$$

- Linex loss function

The linex loss (LL) function is defined in the form as

$$L_{l}\left(\varpi ,\hat{\varpi }\right)=e^{\hbar (\hat{\varpi }-\varpi )}-\hbar \left(\hat{\varpi }-\varpi \right)-1,\;\hbar \neq 0.$$

Under this circumstance, the corresponding Bayes estimator ${\hat{\varpi }}_{l}$ of ϖ can be derived from

$$\varpi_{l}=-\frac{1}{\hbar }E\left(e^{-\hbar \varpi }|\tilde{x}\right).$$

Next, the Bayes estimate $\hat{ℵ}{\left(\mu ,\tau \right)}_{l}$ of ℵ(μ,τ) under LL function is computed by

(14) $$\hat{ℵ}{\left(\mu ,\tau \right)}_{l}=-\frac{1}{\hbar }\log\left[\frac{\int_{0}^{\infty }\int_{0}^{\infty }e^{\hbar ℵ(\mu ,\tau )}\pi_{0}\left(\mu ,\tau \right)L\left(\mu ,\tau |\tilde{x}\right)d\mu \;d\tau }{\int_{0}^{\infty }\int_{0}^{\infty }L\left(\mu ,\tau |\tilde{x}\right)\pi_{0}\left(\mu ,\tau \right)d\mu \;d\tau }\right].$$

Obviously, the Bayes estimatons of unknown parameters μ and τ in three kinds of loss functions cannot be expressed in closed forms. For this reason, we derive the Bayes estimations by the means of Tierney and Kadane method, as well as the importance sampling procedure.

### 4.3. Tierney and Kadane Method

It is hard to reduce the Bayes estimations into closed forms in the shape of the ratio of two integrals. Ref. [19] introduced an alternative method to approximate such ratio of integrals to derive the Bayes estimates of unknown parameters. It is just a simple Taylor approximation around the maximum a posteriori estimate up to second order (also known as saddle-point approximation, see [20]). We regard ℵ(μ,τ) as a function of μ and τ. Then the approximation of Tierney and Kadane approach is summarized as follows.

$$\begin{array}{cc} \lambda (\mu ,\tau ) & =\frac{l\left(\mu ,\tau |X\right)+\log(\pi_{0}\left(\mu ,\tau \right))}{n},\\ \lambda^{*}\left(\mu ,\tau \right) & =\lambda \left(\mu ,\tau \right)+\frac{\log(ℵ(\mu ,\tau ))}{n} \end{array}$$

Based on this method, (11) is re-expressed as

(15) $$\begin{array}{cc} E\left[ℵ\left(\mu ,\tau \right)|\tilde{x}\right]= & \frac{\int_{0}^{\infty }\int_{0}^{\infty }e^{n\lambda^{*}\left(\mu ,\tau \right)}d\mu \;d\tau }{\int_{0}^{\infty }\int_{0}^{\infty }e^{n\lambda (\mu ,\tau )}d\mu \;d\tau }\\ = & \sqrt{\frac{\left|\Lambda_{ℵ}^{*}\right|}{\left|\Lambda \right|}}e^{n[\lambda^{*}\left({\hat{\mu }}_{\lambda^{*}},{\hat{\tau }}_{\lambda^{*}}\right)-\lambda \left({\hat{\mu }}_{\lambda },{\hat{\tau }}_{\lambda }\right)]} \end{array}$$

where $\left({\hat{\mu }}_{\lambda },{\hat{\tau }}_{\lambda }\right)$ and $\left({\hat{\mu }}_{\lambda^{*}},{\hat{\tau }}_{\lambda^{*}}\right)$ are the maximum points of λ(μ,τ) and $\lambda^{*}\left(\mu ,\tau \right)$, respectively. The inverse of the negative Hessian matrix of λ(μ,τ) and $\lambda^{*}\left(\mu ,\tau \right)$ at $\left({\hat{\mu }}_{\lambda },{\hat{\tau }}_{\lambda }\right)$ and $\left({\hat{\mu }}_{\lambda^{*}},{\hat{\tau }}_{\lambda^{*}}\right)$ are denoted as Λ and $\Lambda^{*}$ repectively.

For the truncated normal distribution, we have

(16) $$\begin{array}{cc} \lambda (\mu ,\tau )= & \frac{1}{n}\left\{-\left(c+\frac{3}{2}+\frac{J}{2}\right)\log\left(\tau \right)-\frac{1}{2\tau }\left[b{\left(\mu -a\right)}^{2}+d+\sum_{i=1}^{J}{\left(x_{i}-\mu \right)}^{2}\right]\right.\\ \; & \left.+\sum_{i=1}^{J}R_{i}\log\left(1-\Phi (\frac{x_{i}-\mu }{n})\right)-n\log\left(\Phi (\frac{\mu }{\sqrt{\tau }})\right)-\log\left(\Phi (a\sqrt{\frac{b}{\tau }})\right)+h\left(\mu ,\tau \right)\right\} \end{array}$$

Differentiating (16) with regard to μ and τ so as to obtain ${\hat{\mu }}_{\lambda }$,${\hat{\tau }}_{\lambda }$, we can obtain the following equations

$$\begin{array}{cc} \frac{\partial \lambda }{\partial \mu }= & \frac{1}{n}\left\{-\frac{1}{\tau }\left(\left(b+J\right)\mu -ab-\sum_{i=1}^{J}x_{i}\right)+\sum_{i=1}^{J}\frac{\frac{R_{i}}{\sqrt{\tau }}\phi \left(\eta_{i}\right)}{1-\Phi (\eta_{i})}-\frac{n}{\sqrt{\tau }}\frac{\phi (\eta )}{\Phi (\eta )}+h_{1}\left(\mu ,\tau \right)\right\}=0 \end{array}$$

$$\begin{array}{cc} \frac{\partial \lambda }{\partial \tau }= & \frac{1}{n}\left\{-\frac{c+\frac{3}{2}+\frac{J}{2}}{\tau }+\frac{1}{2\tau^{2}}\left[\sum_{i=1}^{J}{\left(x_{i}-\mu \right)}^{2}+d+b{\left(\mu -a\right)}^{2}\right]+\sum_{i=1}^{J}\frac{R_{i}\eta_{i}}{2\tau }\frac{\phi (\eta_{i})}{1-\Phi (\eta_{i})}\right.\\ \; & \left.\frac{n\mu }{2\tau^{3/2}}\frac{\phi (\eta )}{\Phi (\eta )}+\frac{a\sqrt{b}}{2\tau^{3/2}}\frac{\phi (a\sqrt{\frac{b}{\tau }})}{\Phi (a\sqrt{\frac{b}{\tau }})}+h_{2}\left(\mu ,\tau \right)\right\}=0 \end{array}$$

Thus, we can calculate $\left|\Lambda \right|$, which is derived from

(17) $$\left|\Lambda \right|={\left[\frac{\partial^{2}\lambda }{\partial \mu^{2}}\frac{\partial^{2}\lambda }{\partial \tau^{2}}-\frac{\partial^{2}\lambda }{\partial \mu \partial \tau }\frac{\partial^{2}\lambda }{\partial \tau \partial \mu }\right]}_{\left({\hat{\mu }}_{\lambda },{\hat{\tau }}_{\lambda }\right)}^{-1}$$

where

$$\begin{array}{cc} \frac{\partial^{2}\lambda }{\partial \mu^{2}}= & \frac{1}{n}\left\{-\frac{b+J}{\tau }-\sum_{i=1}^{J}\frac{R_{i}}{\tau }\left[\frac{\phi^{\prime }\left(\eta_{i}\right)}{1-\Phi (\eta_{i})}+{\left(\frac{\phi (\eta_{i})}{1-\Phi (\eta_{i})}\right)}^{2}\right]\right.\\ \; & \left.-\frac{n}{\tau }\left[\frac{\phi^{\prime }\left(\eta \right)}{\Phi (\eta )}-{\left(\frac{\phi (\eta )}{\Phi (\eta )}\right)}^{2}\right]+h_{20}\left(\mu ,\tau \right)\right\} \end{array}$$

$$\begin{array}{cc} \frac{\partial^{2}\lambda }{\partial \mu \partial \tau }= & \frac{1}{n}\left\{\frac{\left(b+J\right)\mu -ab-\sum_{i=1}^{J}x_{i}}{\tau^{2}}-\sum_{i=1}^{J}\frac{R_{i}\eta_{i}}{2\tau^{3/2}}\left[\frac{\phi^{\prime }\left(\eta_{i}\right)}{1-\Phi (\eta_{i})}+{\left(\frac{\phi (\eta_{i})}{1-\Phi (\eta_{i})}\right)}^{2}\right]\right.\\ \; & \left.-\sum_{i=1}^{J}\frac{R_{i}}{2\tau^{3/2}}\frac{\phi (\eta_{i})}{1-\Phi (\eta_{i})}+\frac{n\mu }{4\tau^{2}}\left[\frac{\phi^{\prime }\left(\eta \right)}{\Phi (\eta )}-{\left(\frac{\phi (\eta )}{\Phi (\eta )}\right)}^{2}\right]+\frac{n}{2\tau^{3/2}}\frac{\phi (\eta )}{\Phi (\eta )}+h_{11}\left(\mu ,\tau \right)\right\} \end{array}$$

$$\begin{array}{cc} \frac{\partial^{2}\lambda }{\partial \tau^{2}}= & \frac{1}{n}\left\{\frac{c+\frac{3}{2}+\frac{J}{2}}{\tau^{2}}-\frac{1}{\tau^{3}}\left[b{\left(\mu -a\right)}^{2}+d+\sum_{i=1}^{J}{\left(x_{i}-\mu \right)}^{2}\right]-\sum_{i=1}^{J}\frac{R_{i}\eta_{i}^{2}}{4\tau^{2}}\left[\frac{\phi^{\prime }\left(\eta_{i}\right)}{1-\Phi (\eta_{i})}\right.\right.\\ \; & \left.\left.+{\left(\frac{\phi (\eta_{i})}{1-\Phi (\eta_{i})}\right)}^{2}\right]-\sum_{i=1}^{J}\frac{3R_{i}\eta_{i}}{4\tau^{2}}\frac{\phi (\eta_{i})}{1-\Phi (\eta_{i})}-\frac{n\mu^{2}}{4\tau^{3}}\left[\frac{\phi^{\prime }\left(\eta \right)}{\Phi (\eta )}-{\left(\frac{\phi (\eta )}{\Phi (\eta )}\right)}^{2}\right]\right.\\ \; & \left.-\frac{3n\mu }{4\tau^{5/2}}\frac{\phi (\eta )}{\Phi (\eta )}-\frac{3a\sqrt{b}}{4\tau^{5/2}}\frac{\phi (\frac{a\sqrt{b}}{\sqrt{\tau }})}{\Phi (\frac{a\sqrt{b}}{\sqrt{\tau }})}-\frac{a^{2}b}{4\tau^{3}}\left[\frac{\phi^{\prime }\left(\frac{a\sqrt{b}}{\sqrt{\tau }}\right)}{\Phi (\frac{a\sqrt{b}}{\sqrt{\tau }})}-{\left(\frac{\phi (\frac{a\sqrt{b}}{\sqrt{\tau }})}{\Phi (\frac{a\sqrt{b}}{\sqrt{\tau }})}\right)}^{2}\right]+h_{02}\left(\mu ,\tau \right)\right\} \end{array}$$

Note that $\left|\Lambda_{ℵ}^{*}\right|$ and $\lambda_{ℵ}^{*}$ depend on ℵ(μ,τ).

For the SEL function ℵ(μ,τ)=μ, then the corresponding function $\lambda_{\mu s}^{*}\left(\mu ,\tau \right)$ is obtained as

$$\lambda_{\mu s}^{*}\left(\mu ,\tau \right)=\lambda \left(\mu ,\tau \right)+\frac{\log\mu }{n}$$

In the following, ${\hat{\mu }}_{\lambda^{*}},{\hat{\tau }}_{\lambda^{*}}$ are obtained by solving the following two equations.

$$\frac{\partial \lambda_{\mu s}^{*}}{\partial \mu }=\frac{\partial \lambda }{\partial \mu }+\frac{1}{n\mu }=0,\;\frac{\partial \lambda_{\mu s}^{*}}{\partial \tau }=\frac{\partial \lambda }{\partial \tau }=0$$

Then we have $\left|\Lambda_{\mu s}^{*}\right|$, which is given by

$$\left|\Lambda_{\mu s}^{*}\right|={\left[\frac{\partial^{2}\lambda_{\mu s}^{*}}{\partial \mu^{2}}\frac{\partial^{2}\lambda_{\mu s}^{*}}{\partial \tau^{2}}-\frac{\partial^{2}\lambda_{\mu s}^{*}}{\partial \mu \partial \tau }\frac{\partial^{2}\lambda_{\mu s}^{*}}{\partial \tau \partial \mu }\right]}^{-1}$$

where

$$\frac{\partial^{2}\lambda_{\mu s}^{*}}{\partial \mu^{2}}=\frac{\partial^{2}\lambda }{\partial \mu^{2}}-\frac{1}{n\mu^{2}},\;\frac{\partial^{2}\lambda_{\mu s}^{*}}{\partial \tau \partial \mu }=\frac{\partial^{2}\lambda_{\mu s}^{*}}{\partial \mu \partial \tau }=\frac{\partial^{2}\lambda }{\partial \mu \partial \tau },\;\frac{\partial^{2}\lambda_{\mu s}^{*}}{\partial \tau^{2}}=\frac{\partial^{2}\lambda }{\partial \tau^{2}}.$$

Using the equations above, the Bayes estimator of μ comes to be

$${\hat{\mu }}_{s}=\sqrt{\frac{\left|\Lambda_{\mu s}^{*}\right|}{\left|\Lambda \right|}}e^{n[\lambda_{\mu s}^{*}\left({\hat{\mu }}_{\lambda^{*}},{\hat{\tau }}_{\lambda^{*}}\right)-\lambda \left({\hat{\mu }}_{\lambda },{\hat{\tau }}_{\lambda }\right)]}$$

Going through a similar process, under the same loss function the Bayesian estimator of τ is achieved.

As for the GEL function, we consider $ℵ\left(\mu ,\tau \right)=\mu^{-q}$ for μ and then the corresponding function $\lambda_{\mu e}^{*}\left(\mu ,\tau \right)$ is expressed as

$$\lambda_{\mu e}^{*}\left(\mu ,\tau \right)=\lambda \left(\mu ,\tau \right)-\frac{q\log\mu }{n}$$

Subsequently, the following equations are solved to obtain $\left({\hat{\mu }}_{\lambda^{*}},{\hat{\tau }}_{\lambda^{*}}\right)$

$$\frac{\partial \lambda_{\mu e}^{*}}{\partial \mu }=\frac{\partial \lambda }{\partial \mu }-\frac{q}{n\mu }=0,\;\frac{\partial \lambda_{\mu e}^{*}}{\partial \tau }=\frac{\partial \lambda }{\partial \tau }=0$$

Then, we have $\left|\Lambda_{\mu e}^{*}\right|$, which is given by

$$\left|\Lambda_{\mu e}^{*}\right|={\left[\frac{\partial^{2}\lambda_{\mu e}^{*}}{\partial \mu^{2}}\frac{\partial^{2}\lambda_{\mu e}^{*}}{\partial \tau^{2}}-\frac{\partial^{2}\lambda_{\mu e}^{*}}{\partial \mu \partial \tau }\frac{\partial^{2}\lambda_{\mu e}^{*}}{\partial \tau \partial \mu }\right]}^{-1}$$

where

$$\frac{\partial^{2}\lambda_{\mu e}^{*}}{\partial \mu^{2}}=\frac{\partial^{2}\lambda }{\partial \mu^{2}}+\frac{q}{n\mu^{2}},\;\frac{\partial^{2}\lambda_{\mu e}^{*}}{\partial \tau \partial \mu }=\frac{\partial^{2}\lambda_{\mu e}^{*}}{\partial \mu \partial \tau }=\frac{\partial^{2}\lambda }{\partial \mu \partial \tau },\;\frac{\partial^{2}\lambda_{\mu e}^{*}}{\partial \tau^{2}}=\frac{\partial^{2}\lambda }{\partial \tau^{2}}.$$

After that, the Bayes estimator of μ is

$${\hat{\mu }}_{e}=\sqrt{\frac{\left|\Lambda_{\mu e}^{*}\right|}{\left|\Lambda \right|}}e^{-\frac{n}{q}\left[\lambda_{\mu e}^{*}\left({\hat{\mu }}_{\lambda^{*}},{\hat{\tau }}_{\lambda^{*}}\right)-\lambda \left({\hat{\mu }}_{\lambda },{\hat{\tau }}_{\lambda }\right)\right]}$$

Likewise, the Bayesian estimator of τ is realized based on this loss function.

When it comes to the LL function, $ℵ\left(\mu ,\tau \right)=e^{\hbar \mu }$ for μ is under consideration and then the corresponding function $\lambda_{\mu l}^{*}\left(\mu ,\tau \right)$ is given by

$$\lambda_{\mu l}^{*}\left(\mu ,\tau \right)=\lambda \left(\mu ,\tau \right)-\frac{\hbar \mu }{n}$$

Later, ${\hat{\mu }}_{\lambda^{*}}$ and ${\hat{\tau }}_{\lambda^{*}}$ are derived by solving the following equations

$$\frac{\partial \lambda_{\mu l}^{*}}{\partial \mu }=\frac{\partial \lambda }{\partial \mu }-\frac{\hbar }{n}=0,\;\frac{\partial \lambda_{\mu l}^{*}}{\partial \tau }=\frac{\partial \lambda }{\partial \tau }=0$$

Then, we have $\left|\Lambda_{\mu l}^{*}\right|$, which is given by

$$\left|\Lambda_{\mu l}^{*}\right|={\left[\frac{\partial^{2}\lambda_{\mu l}^{*}}{\partial \mu^{2}}\frac{\partial^{2}\lambda_{\mu l}^{*}}{\partial \tau^{2}}-\frac{\partial^{2}\lambda_{\mu l}^{*}}{\partial \mu \partial \tau }\frac{\partial^{2}\lambda_{\mu l}^{*}}{\partial \tau \partial \mu }\right]}^{-1}$$

where

$$\frac{\partial^{2}\lambda_{\mu l}^{*}}{\partial \mu^{2}}=\frac{\partial^{2}\lambda }{\partial \mu^{2}},\;\frac{\partial^{2}\lambda_{\mu l}^{*}}{\partial \tau \partial \mu }=\frac{\partial^{2}\lambda_{\mu l}^{*}}{\partial \mu \partial \tau }=\frac{\partial^{2}\lambda }{\partial \mu \partial \tau },\;\frac{\partial^{2}\lambda_{\mu l}^{*}}{\partial \tau^{2}}=\frac{\partial^{2}\lambda }{\partial \tau^{2}}.$$

The Bayes estimator of μ turns into

$${\hat{\mu }}_{l}=-\frac{1}{\hbar }\log\left\{\sqrt{\frac{\left|\Lambda_{\mu l}^{*}\right|}{\left|\Lambda \right|}}e^{n[\lambda_{\mu l}^{*}\left({\hat{\mu }}_{\lambda^{*}},{\hat{\tau }}_{\lambda^{*}}\right)-\lambda \left({\hat{\mu }}_{\lambda },{\hat{\tau }}_{\lambda }\right)]}\right\}$$

Similarly, under the LL function, the Bayesian estimator of τ can be attained.

### 4.4. Importance Sampling Procedure

Since the T-K method fails to develop the interval estimators, an importance sampling procedure is proposed to construct Bayesian credible intervals in this part. The importance sampling procedure is an effective approach to attain Bayes estimates for TN(μ,τ). In the meanwhile, the HPD intervals can be constructed through this method under generalized progressive hybrid censored data. Recall that the posterior distribution of μ and τ for μ > 0, τ > 0 is the following form

(18) $$\begin{array}{cc} \pi (\mu ,\tau |\tilde{x})\propto & \frac{{\left[\Phi \left(\frac{\mu }{\sqrt{\tau }}\right)\right]}^{-n}}{\Phi \left(\frac{a\sqrt{b}}{\sqrt{\tau }}\right)}{\left(\frac{1}{\tau }\right)}^{(c+\frac{1+J}{2})+1}e^{-\frac{1}{2\tau }\left[d+\sum_{i=1}^{J}{\left(x_{i}-\mu \right)}^{2}+b{\left(\mu -a\right)}^{2}\right]}\\ \; & \times \prod_{i=1}^{J}{\left(1-\Phi \left(\frac{x_{i}-\mu }{\sqrt{\tau }}\right)\right)}^{R_{i}}H\left(\mu ,\tau \right). \end{array}$$

After some calculations, (18) is reduced to

(19) $$\begin{array}{cc} \pi (\mu ,\tau |\tilde{x})\propto & IG_{\tau }\left(c+\frac{J}{2},\frac{1}{2}\left(\sum_{i=1}^{J}x_{i}+a^{2}b+d-\frac{{\left(ab+\sum_{i=1}^{J}x_{i}\right)}^{2}}{b+J}\right)\right)\\ \; & TN_{\mu |\tau }\left(\frac{ab+\sum_{i=1}^{J}x_{i}}{b+J},\frac{\tau }{b+J}\right)W\left(\mu ,\tau \right) \end{array}$$

where

$$W\left(\mu ,\tau \right)=H\left(\mu ,\tau \right)\prod_{i=1}^{J}{\left(1-\Phi \left(\frac{x_{i}-\mu }{\sqrt{\tau }}\right)\right)}^{R_{i}}\Phi \left(\frac{ab+\sum_{i=1}^{J}x_{i}}{\sqrt{\tau (b+J)}}\right)\frac{{\left[\Phi \left(\frac{\mu }{\sqrt{\tau }}\right)\right]}^{-n}}{\Phi \left(\frac{a\sqrt{b}}{\sqrt{\tau }}\right)}$$

Through the importance sampling procedure, we attain the Bayes estimates of μ and τ. The importance sampling procedure method is briefly described as follows:

Step 1: Generate $\tau_{1}$ from $IG_{\tau }\left(c+\frac{J}{2},\frac{1}{2}\left(\sum_{i=1}^{J}x_{i}+a^{2}b+d-\frac{{\left(ab+\sum_{i=1}^{J}x_{i}\right)}^{2}}{b+J}\right)\right)$;
Step 2: Sample $\mu_{1}$ randomly from $TN_{\mu |\tau }\left(\frac{ab+\sum_{i=1}^{J}x_{i}}{b+J},\frac{\tau }{b+J}\right)$;
Step 3: Perform Step 1 and Step 2, *k* times to obtain $\left(\mu_{1},\tau_{1}\right)$, $\left(\mu_{2},\tau_{2}\right)$, ⋯, $\left(\mu_{k},\tau_{k}\right)$
Step 4: Now the Bayes estimation of ℵ(μ,τ) can be derived as follows.
  $${\hat{ℵ}}_{IS}=\frac{\sum_{i=1}^{k}ℵ\left(\mu_{i},\tau_{i}\right)W\left(\mu_{i},\tau_{i}\right)}{\sum_{i=1}^{k}W\left(\mu_{i},\tau_{i}\right)}$$

Furthermore, the method introduced in [21] is applied to derive the 100(1−ζ)% Bayesian credible intervals for the given truncated normal distribution. Assume that 0 <ζ< 1 and ${ℵ}_{\zeta }$ satisfies $P(ℵ\left(\mu ,\tau \right)\leq {ℵ}_{\zeta })=\zeta$. For a prefixed ζ, we attain the estimation of ${ℵ}_{\zeta }$ and use it to establish the HPD intervals for ℵ(μ,τ).

First, we substitute $ℵ(\mu_{i},\tau_{i})$ with ${ℵ}_{i}$ for simplicity and suppose

(20) $$\omega_{i}=\frac{W(\mu_{i},\tau_{i})}{\sum_{i=1}^{k}W\left(\mu_{i},\tau_{i}\right)},\;\;i=1,\cdots ,k.$$

Sort $\left\{\left({ℵ}_{1},\omega_{1}\right),\cdots ,\left({ℵ}_{k},\omega_{k}\right)\right\}$ into $\left\{\left({ℵ}_{\left(1\right)},\omega_{\left(1\right)}\right),\cdots ,\left({ℵ}_{\left(k\right)},\omega_{\left(k\right)}\right)\right\}$, where ${ℵ}_{\left(1\right)}$ < ⋯ < ${ℵ}_{\left(k\right)}$ and ${ℵ}_{\left(i\right)}$ is connected with $\omega_{i}$ for i=1,⋯,k. Then the estimator of ${ℵ}_{\zeta }$ is $\hat{{ℵ}_{\zeta }}={ℵ}_{\left(z_{\zeta }\right)}$, where $z_{\zeta }$ is an integer satisfying

(21) $$\sum_{i=1}^{z_{\zeta }}\omega_{\left(i\right)}\leq \zeta \leq \sum_{i=1}^{z_{\zeta }+1}\omega_{\left(i\right)}$$

Hence, a 100(1−ζ)% Bayesian credible interval of ℵ(μ,τ) can be obtained by ($\hat{{ℵ}_{\upsilon }}$, $\hat{{ℵ}_{\upsilon +1-\zeta }}$) for $\nu =\omega_{\left(1\right)}$, $\omega_{\left(1\right)}+\omega_{\left(2\right)}$, ⋯, $\sum_{i=1}^{z_{\left(1-\zeta \right)}}\omega_{\left(i\right)}$. Eventually, the 100(1−ζ)% HPD interval of ℵ(μ,τ) is obtained by (${\hat{ℵ}}_{\nu^{*}}$, ${\hat{ℵ}}_{\nu^{*}+1-\zeta }$) where $\nu^{*}$ satisafying

(22) $$\hat{{ℵ}_{\nu^{*}+1-\zeta }}-\hat{{ℵ}_{\nu^{*}}}\leq \hat{{ℵ}_{\nu +1-\zeta }}-\hat{{ℵ}_{\nu }}$$

for all ν.

## 5. Simulation Study

### 5.1. Simulation

In attempts to analyze the performance of different methods introduced in previous sections, we utilize R software to conduct the simulation experiments. In light of the algorithm proposed in [22], a progressive type-II censored sample under any continuous distribution can be generated. By adopting this method, we can generate a generalized progressive hybrid censoring sample that is subject to the truncated normal distribution. Please see Algorithm 1.
**Algorithm 1** Generate a generalized progressive hybrid censoring sample from truncated normal distribution.1:Pre-assign *n*,*m* and the censoring scheme $\left(R_{1},R_{2},\cdots ,R_{m}\right)$.2:Generate *m* independent observations $W_{1},W_{2},\cdots ,W_{m}$, all of which are subject to the standard uniform distribution U(0,1).3:For pre-fixed censoring scheme, we compute $V_{i}$ = $W_{i}^{1/(i+\sum_{j=(m-i+1)}^{m}R_{j})}$, i=1,2,⋯,m.4:Set $U_{i}=1-\prod_{j=m-i+1}^{m}V_{j}$, for i=1,2,⋯,m. Then $U_{1},U_{2},\cdots ,U_{m}$ is a progressive Type-II censored sample with size *m* from a uniform distribution from zero to one.5:For known values of parameters μ and τ, the desired progressive Type-II censored sample out of the truncated normal distribution TN(μ,τ), is $X_{i}=\mu +\sqrt{\tau }\Phi^{-1}\left(1-\left(1-U_{i}\right)\Phi \left(\frac{\mu }{\sqrt{\tau }}\right)\right)$, for i=1,2,⋯,m, where $\Phi^{-1}$ is the inverse cumulative distribution function of truncated normal distribution.6:If $T<X_{k}<X_{m}$, the generalized progressive hybrid censored sample is $X=(X_{1},X_{2},\cdots ,X_{k})$.7:If $X_{k}<T<X_{m}$, *J* can be obtained which satisfies $X_{J}<T<X_{J+1}$, the generalized progressive hybrid censored sample is $\left(X_{1},X_{2},\cdots ,X_{J}\right)$.8:If $X_{k}<X_{m}<T$, the generalized progressive hybrid censoring sample is $\left(X_{1},X_{2},\cdots ,X_{m}\right)$.

Without loss of generality, we take μ = 0.5, τ = 1 for diverse values of *T*, *n*, *m* and *k* to generate a generalized progressive hybrid censored sample from the left-truncated normal distribution when truncation point is zero. Meanwhile, two kinds of censoring schemes are considered, which are:

Scheme I: $R_{m}=n-m$, $R_{i}=0,i=1,\cdots ,m-1$.

Scheme II: $R_{1}=n-m$, $R_{i}=0,i=2,\cdots ,m$.

In point estimation, we compute the MLEs and Bayes estimates. For MLEs, the EM estimation method is employed to calculate the MLEs of μ and τ, where the *nleqslv* package with Broyden method in R software is applied to solve the nonlinear equations in the maximization step. Besides, the MLEs are derived by the N-R approach for comparative purposes. This method can be achieved by the function ‘optim’ in R software. Here the true values of parameters are considered as the initial values for the N-R method and the EM algorithm. The value of the tolerance limit ε is 0.0001 for all simulations. Table A1 and Table A2, shown in the Appendix A, compare the results of the EM and N-R methods in terms of average absolute biases (ABs), the associated mean squared errors (MSEs) and average number of iterations (AIs) until convergence. They are

$$ABs=\frac{1}{N}\sum_{i=1}^{N}\left|{\hat{\varpi }}_{i}-\varpi \right|$$

$$MSEs=\frac{1}{N}\sum_{i=1}^{N}{\left({\hat{\varpi }}_{i}-\varpi \right)}^{2}.$$

where *N* stands for the simulation times, ϖ is the true value, ${\hat{\varpi }}_{i}$ denotes the *i*-th estimate of ϖ.

Besides, the Bayesian estimates against diverse loss functions including SEL, LL, and GEL functions are obtained by the means of the T-K method and importance sampling procedure. In order to appropriate true values better, the values of hyper-parameters a=0.05, b=1, c=8, d=0.3 in prior distributions might be a wise choice based on numerous experimental simulations. The desired estimates are obtained with *ℏ* = 0.35, 0.45 for the linex loss function. The general entropy loss function is considered with q=0.8 and q=1.1 to calculate the relative estimates. The ABs and MSEs are derived to evaluate the accuracy of the estimations. All the estimates are derived by replicating 1000 times in each case. The simulation results, Table A3 and Table A4, are shown in the Appendix A.

From these tables, some conclusions can be drawn:

(1) There is no significant difference between the EM algorithm and N-R algorithm in terms of ABs and MSEs.
(2) The N-R method takes fewer steps until convergence than the EM.
(3) The results of ℏ=0.35 show a bit more precise than those of ℏ=0.45 for the linex loss function.
(4) q=0.8 is a wiser choice than q=1.1 when the general entropy loss function is under consideration.
(5) Set *T*, *n*, *m* and *k* invariant, Scheme II in which the censored units happen when the first failure is observed shows a more precise estimate than Scheme I for most cases.
(6) Between Bayes estimation methods, neither the TK method nor the importance sampling technique performs consistently better since in some cases T-K estimates behave better and in some cases important sampling estimates perform better.
(7) When the sample size *n* increases, the ABs of all estimates show downward trends.
(8) When *T*, *n* and *m* keep invariable, the behaviors of both MLEs and Bayes estimates become better concerning the values of MSEs and ABs with the larger values of *k*.
(9) When *k*, *n* and *m* keep invariable, the ABs and MSEs of all estimates fluctuate slightly, and the tendency is not significant with the growth of *T*.
(10) When *T*, *n* and *k* keep invariable, both MLEs and Bayes estimates tend to have smaller MSEs with the larger values of *m*.
(11) Overall, the Bayes estimates seem to be marginally better compared to the MLEs.

In interval estimation, we derive 95% confidence intervals (CIs) of parameters using the MLEs, log-transformed MLEs, Boot-p approach and 95% HPD intervals for Scheme I and Scheme II. We compare the coverage probabilities (CPs) and average lengths (ALs) of these interval estimates. The simulation results are reported in Table A5 and Table A6, shown in the Appendix A.

From these table, some conclusions are summarized as follows.

(1) For confidence intervals, the Log-CIs perform much better than the ACIs in the sense of having higher coverage probabilities.
(2) When the sample size *n* gets larger, the CPs of all interval estimates tend to decrease.
(3) Boot-p CIs show higher coverage probabilities and narrower interval lengths than the ACIs and Log-CIs when the sample size is small.
(4) With *n*, *m*, and *k* keeping invariant, the CPs and ALs of all estimates fluctuate slightly and the tendency is not significant with an increase of *T*.
(5) The HPD intervals are slightly better than other interval estimates based on the CPs.
(6) Scheme II usually performs better than Scheme I with regard to the CPs.

### 5.2. Real Data Analysis

**Figure 3:**
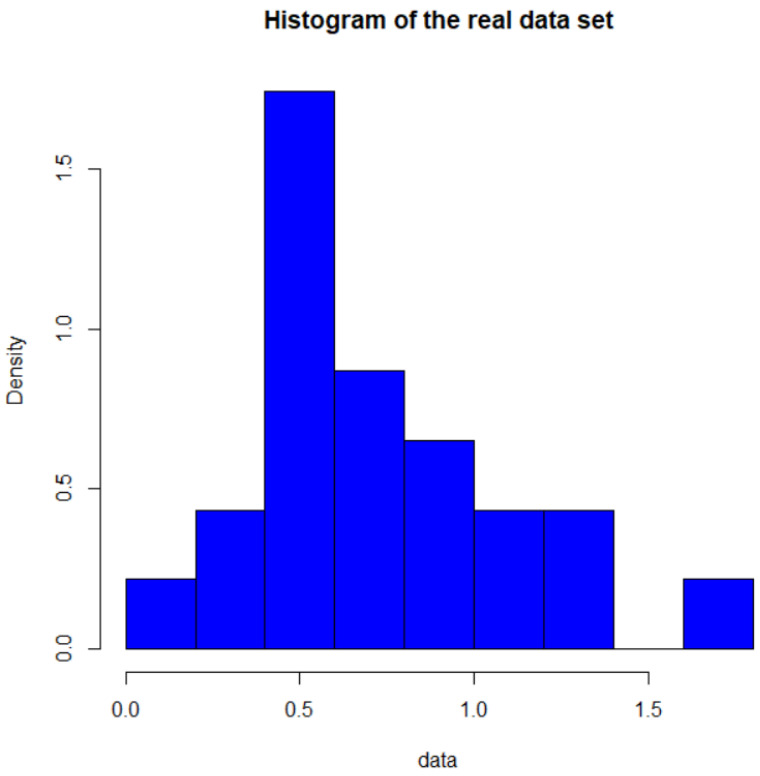
Histogram of the real data set.

One authentic example has been considered to demonstrate the performance of the proposed estimation approaches in this section. The data set is obtained from [23] (Lawless 1982, page 288), which shows the number of million revolutions before 23 ball bearings had failed. The data are given in Table 1 and shown as a histogram in Figure 3.

Prior to analyzing the example, one question that may arise is whether the data set comes from the truncated normal distribution. In order to validate the hypothesis, we fit the truncated normal distribution to the data set, competing with folded normal distribution (FN) and half-normal distribution (HN). The associated probability density functions of FN and HN for *x* > 0 are respectively written as follows.

$$f\left(x;\mu ,\tau \right)=\frac{e^{-\frac{{\left(x-\mu \right)}^{2}}{2\tau }}+e^{-\frac{{\left(x+\mu \right)}^{2}}{2\tau }}}{\sqrt{2\pi \tau }},\;x>0;\;\mu >0,\;\tau >0.$$

and

$$f\left(x;\tau \right)=\sqrt{\frac{2}{\pi \tau }}e^{-\frac{x^{2}}{2\tau }},\;x>0;\;\tau >0.$$

In order to assess the goodness of fit of these given models, we take advantage of −log(L), and Kolmogorov-Smirnov (K-S) statistic, defined by $D_{n}=\underset{x}{\sup}\left|F_{n}\left(x\right)-F\left(x\right)\right|$, where *L* is the maximum of the likelihood function, *n* is the number of observations, $F_{n}\left(x\right)$ is the cumulative distribution function of the sample set, and F(x) is the assumed cumulative distribution function. These estimated values are shown in Table 2. Under the complete data, the classical estimates of these given distributions are obtained additionally. In view of the fact that the truncated normal distribution has the lowest values for K-S and −log(L) statistics, it is reasonable to say we have no proof leading to rejection of the null hypothesis.

Then the following generalized progressive hybrid censored samples can be generated by setting m=18, and $R_{1}=5$, $R_{2}=\cdots =R_{18}=0$. In this example, we take T=1, k=16 for Case I, T=1, k=12 for Case II, as well as T=1.8, k=12 for Case III.

Table 3 presents the exact values of point estimations of μ and τ under the generalized progressive hybrid censored sample. The N-R algorithm and the EM algorithm are employed to derive the MLEs of parameters of the truncated normal distribution. We take the estimates under the complete data for initializing these two methods. For Bayes estimations, non-informative prior distributions, a=b=c=d=0, are applied to compute the exact values under symmetric and asymmetric loss functions based on T-K approximation and importance sampling procedure. We choose q=0.8 and q=1.1 for general entropy loss function, and fix ℏ=0.35 and ℏ=0.45 for the linex loss function. Table 4 shows the results of the 95% CIs and HPD intervals of unknown parameters μ and τ. As can be seen from the tables, one can notice that the results of MLEs, Bayes estimates under SEL function and importance sampling procedure are quite close to each other.

## 6. Conclusive Remarks

In the paper, we address the problem of making statistical inference for the truncated normal distribution when the truncation point is zero with the generalized progressive hybrid censored data. The N-R and EM algorithms are applied to calculate the MLEs of unknown parameters. The associated estimates are computed through numerous simulations by taking the true unknown parameters as an initial guess. Further, making use of the asymptotic normality property of the maximum likelihood estimator, we construct the 95% ACIs as well. As for the small sample size, Boot-p method is suggested to develop the Boot-p intervals. Considering different kinds of loss functions, we derive Bayes estimates through T-K approximation and the importance sampling procedure. The latter one is also employed to construct the Bayesian credible intervals. Extensive simulations are conducted to examine how the proposed approaches work. It is notable that if proper prior information is available on unknown parameters then corresponding Bayes estimates are superior to respective MLEs. Among intervals, Bayesian credible intervals are slightly better than other invertals. One authentic example is studied for illustrative purposes. The considered model is found to be suitable for this case and the proposed approaches perform well.

While we consider the statistical inference of the truncated normal distribution at zero in this paper, the proposed approaches can be extended to the doubly truncated normal distribution and the singly truncated normal distribution at any fixed truncation point. The statistical inference on the doubly truncated distribution with unknown truncation points in [24] provides a good starting point for discussion and further research. Extensive work needs to be carried out in this direction.

## Figures and Tables

**Table 1:** The number of revolutions (in millions) before failure of 23 ball bearings.

0.1788	0.2892	0.3300	0.4152	0.4212	0.4560	0.4840	0.5184
0.5196	0.5412	0.5556	0.6780	0.6864	0.6864	0.6888	0.8412
0.9312	0.9864	1.0512	1.0584	1.2792	1.2804	1.7340	

**Table 2:** Estimated values of various criteria for different distributions.

Distribution	μ	τ	−log(L)	K-S
TN	0.68079	0.16436	8.80069	0.16832
FN	0.71401	0.14623	9.26571	0.17858
HN	-	0.65601	11.84542	0.26135

**Table 3:** Point estimations of the real dataset.

Case	Parameter	MLE	MLE	SEL	GEL		LL		IS
		(N-R)	(EM)		*q* = 0.8	*q* = 1.1	*ℏ* = 0.35	*ℏ* = 0.45	
I	μ	0.80462	0.72634	0.71307	0.64764	0.55900	1.88966	1.88966	0.78965
	τ	0.12125	0.14501	0.09444	0.09462	0.04257	1.11628	1.16278	0.14589
II	μ	0.78957	0.71985	0.69074	0.81490	0.75648	1.31132	1.41716	0.75745
	τ	0.09959	0.13672	0.11179	0.22141	0.13891	1.04535	1.05878	0.10030
III	μ	0.80895	0.73029	0.75061	0.79298	0.73061	1.29832	1.39946	0.79024
	τ	0.13780	0.16331	0.24271	0.30696	0.21909	1.06950	1.09043	0.17267

**Table 4:** Interval estimations of the real dataset.

Case	Parameter	ACIs	Log-CIs	HPD Intervals	Boot-p CIs
I	μ	(0.63866, 0.97059)	(0.63854, 0.98013)	(0.64351, 0.95027)	(0.63901, 0.96593)
	τ	(0.02390, 0.21861)	(0.02407, 0.21534)	(0.02519, 0.20918)	(0.02432, 0.21568)
II	μ	(0.63542, 0.94373)	(0.62853, 0.93851)	(0.64864, 0.93061)	(0.63854, 0.94837)
	τ	(0.02336, 0.18983)	(0.02161, 0.17734)	(0.02053, 0.17918)	(0.02241, 0.17782)
III	μ	(0.63058, 0.98731)	(0.63509, 0.98602)	(0.63837, 0.98069)	(0.64013, 0.98165)
	τ	(0.03544, 0.24016)	(0.03641, 0.24505)	(0.04068, 0.24033)	(0.03871, 0.24186)

## Appendix A. Simulation Results

**Table A1:** Comparisons between EM and N-R algorithms with μ = 0.5, τ=1 when *T* = 0.4.

*n*	*m*	*k*	Sch	Method	ABs(μ)	ABs(τ)	MSEs(μ)	MSEs(τ)	AIs
30	20	16	I	EM	0.1766	0.3179	0.0503	0.1563	21.35
				N-R	0.1708	0.3003	0.0455	0.1385	7.39
			II	EM	0.1737	0.3053	0.0473	0.1459	21.03
				N-R	0.1814	0.2723	0.0530	0.1168	6.73
		18	I	EM	0.1641	0.2802	0.0423	0.1254	20.30
				N-R	0.1644	0.2957	0.0421	0.1349	7.11
			II	EM	0.1642	0.2957	0.0434	0.1298	20.57
				N-R	0.1786	0.2654	0.0508	0.1099	6.43
30	25	16	I	EM	0.1717	0.2992	0.0458	0.1312	21.35
				N-R	0.1732	0.3073	0.0477	0.1459	7.43
			II	EM	0.1696	0.3160	0.0453	0.1520	21.37
				N-R	0.1718	0.2790	0.0466	0.1220	6.96
		18	I	EM	0.1672	0.2885	0.0438	0.1253	20.68
				N-R	0.1627	0.2957	0.0420	0.1349	7.13
			II	EM	0.1614	0.2929	0.0417	0.1324	20.81
				N-R	0.1713	0.2779	0.0453	0.1168	6.79
60	50	38	I	EM	0.1151	0.2035	0.0205	0.0649	19.08
				N-R	0.1152	0.1991	0.0203	0.0614	6.58
			II	EM	0.1159	0.2004	0.0213	0.0616	18.85
				N-R	0.1220	0.1856	0.0230	0.0552	6.27
		42	I	EM	0.1129	0.1854	0.0196	0.0538	18.58
				N-R	0.1092	0.1809	0.0189	0.0529	6.30
			II	EM	0.1090	0.1862	0.0184	0.0534	18.73
				N-R	0.1228	0.1847	0.0234	0.0531	6.12
60	55	38	I	EM	0.1138	0.1945	0.0207	0.0595	18.99
				N-R	0.1168	0.1964	0.0218	0.0597	6.54
			II	EM	0.1182	0.2081	0.0220	0.0666	19.14
				N-R	0.1080	0.1779	0.0185	0.0497	6.17
		42	I	EM	0.1089	0.1867	0.0185	0.0539	18.63
				N-R	0.1095	0.1864	0.0192	0.0552	6.35
			II	EM	0.1043	0.1820	0.0173	0.0502	18.62
				N-R	0.1099	0.1802	0.0195	0.0502	6.17
100	80	62	I	EM	0.0893	0.1575	0.0123	0.0384	18.21
				N-R	0.0893	0.1549	0.0126	0.0374	6.25
			II	EM	0.0889	0.1536	0.0125	0.0367	18.14
				N-R	0.0978	0.1505	0.0148	0.0349	5.99
		70	I	EM	0.0837	0.1463	0.0109	0.0338	17.87
				N-R	0.0852	0.1452	0.0115	0.0332	6.11
			II	EM	0.0851	0.1456	0.0114	0.0329	18.05
				N-R	0.0911	0.1399	0.0128	0.0313	5.84
100	90	62	I	EM	0.0911	0.1547	0.0132	0.3666	18.29
				N-R	0.0883	0.1547	0.0124	0.0367	6.27
			II	EM	0.0851	0.1563	0.0112	0.0386	18.17
				N-R	0.0902	0.1504	0.0126	0.036	6.07
		70	I	EM	0.0872	0.1435	0.0118	0.0315	17.92
				N-R	0.0834	0.1424	0.0108	0.0316	6.1
			II	EM	0.0818	0.1462	0.0109	0.0334	17.83
				N-R	0.0869	0.1373	0.0119	0.0303	5.95

**Table A2:** Comparisons between EM and N-R algorithms with μ = 0.5, τ=1 when *T* = 0.6.

*n*	*m*	*k*	Sch	Method	ABs(μ)	ABs(τ)	MSEs(μ)	MSEs(τ)	AIs
30	20	16	I	EM	0.1744	0.3039	0.0478	0.1360	21.60
				N-R	0.1719	0.3043	0.0459	0.1435	7.37
			II	EM	0.1739	0.3064	0.0476	0.1440	20.76
				N-R	0.1857	0.2737	0.0552	0.1174	6.75
		18	I	EM	0.1648	0.2685	0.0432	0.1102	20.25
				N-R	0.1671	0.3006	0.0436	0.1402	7.18
			II	EM	0.1698	0.2952	0.0445	0.1367	20.79
				N-R	0.1828	0.2553	0.0522	0.1001	6.44
30	25	16	I	EM	0.1748	0.3159	0.0487	0.1516	21.17
				N-R	0.1639	0.3088	0.0427	0.1442	7.41
			II	EM	0.1796	0.3119	0.0513	0.1538	21.24
				N-R	0.1678	0.2768	0.0438	0.1175	6.76
		18	I	EM	0.1678	0.2854	0.0432	0.1288	20.42
				N-R	0.1687	0.2865	0.0436	0.1294	6.78
			II	EM	0.1614	0.2929	0.0417	0.1324	20.81
				N-R	0.1713	0.2779	0.0453	0.1168	6.79
60	50	38	I	EM	0.1119	0.1979	0.0199	0.0601	18.95
				N-R	0.1125	0.2035	0.0199	0.0633	6.61
			II	EM	0.1127	0.1925	0.0201	0.0570	18.72
				N-R	0.1219	0.1864	0.0231	0.0553	6.30
		42	I	EM	0.1088	0.1866	0.0187	0.0548	18.46
				N-R	0.1101	0.1833	0.0186	0.0521	6.32
			II	EM	0.1098	0.1846	0.0184	0.0545	18.53
				N-R	0.1227	0.1756	0.0229	0.0493	6.06
60	55	38	I	EM	0.1127	0.1922	0.0196	0.0562	18.76
				N-R	0.1173	0.2017	0.0217	0.0628	6.59
			II	EM	0.1152	0.1999	0.0205	0.0624	18.99
				N-R	0.1187	0.1869	0.0220	0.0544	6.44
		42	I	EM	0.1094	0.1857	0.0183	0.0541	18.50
				N-R	0.1115	0.1832	0.0199	0.0516	6.30
			II	EM	0.1127	0.1872	0.0197	0.0562	19.01
				N-R	0.1129	0.1842	0.0198	0.0542	6.21
100	80	62	I	EM	0.0903	0.1557	0.0131	0.0379	18.12
				N-R	0.0873	0.1582	0.0122	0.0382	6.28
			II	EM	0.0876	0.1603	0.0121	0.0401	18.36
				N-R	0.0950	0.1540	0.0141	0.0374	6.06
		70	I	EM	0.0864	0.1454	0.0116	0.0325	17.88
				N-R	0.0857	0.1396	0.0118	0.0309	6.10
			II	EM	0.0898	0.1416	0.0125	0.0317	17.80
				N-R	0.0900	0.1331	0.0128	0.0280	5.81
100	90	62	I	EM	0.0934	0.1513	0.0135	0.0362	17.99
				N-R	0.0885	0.1581	0.0124	0.0398	6.26
			II	EM	0.0839	0.1527	0.0110	0.0370	18.09
				N-R	0.0930	0.1496	0.0136	0.0347	6.14
		70	I	EM	0.0871	0.1421	0.0118	0.0313	17.67
				N-R	0.0861	0.1472	0.0163	0.0335	6.12
			II	EM	0.0826	0.1393	0.0108	0.0308	17.81
				N-R	0.0836	0.1404	0.0111	0.0312	5.95

**Table A3:** ABs and MSEs (within bracket) of different estimates with μ = 0.5, τ = 1 when *T* = 0.4.

*n*	*m*	*k*	Sch	Par	SEL	LL		GEL		IS
						*ℏ* = 0.35	*ℏ* = 0.45	*q* = 0.8	*q* = 1.1	
30	20	16	I	μ	0.7893	0.1661	0.3953	0.1674	0.1782	0.0951
					(0.6679)	(0.0398)	(0.2963)	(0.0646)	(0.0775)	(0.0028)
				τ	0.1953	0.1530	0.1961	0.0811	0.3620	0.1523
					(0.0496)	(0.0530)	(0.0403)	(0.0127)	(0.1515)	(0.0157)
			II	μ	0.7730	0.1647	0.3324	0.1504	0.1873	0.0947
					(0.6410)	(0.0365)	(0.0915)	(0.0533)	(0.0802)	(0.0072)
				τ	0.1677	0.1529	0.1961	0.0984	0.2404	0.1505
					(0.0553)	(0.0328)	(0.0402)	(0.0191)	(0.0718)	(0.0153)
		18	I	μ	0.7662	0.1627	0.3800	0.1423	0.1709	0.0942
					(0.6308)	(0.0856)	(0.2318)	(0.0275)	(0.0350)	0.0029
				τ	0.1710	0.1719	0.1848	0.0730	0.0906	0.1570
					(0.0398)	(0.0237)	(0.0357)	(0.0144)	(0.0158)	(0.0126)
			II	μ	0.6588	0.1476	0.2354	0.1422	0.1705	0.0891
					(0.4745)	(0.0304)	(0.1213)	(0.0271)	(0.0339)	(0.0022)
				τ	0.1563	0.1449	0.1838	0.0698	0.0412	0.1525
					(0.0384)	(0.0283)	(0.0322)	(0.0138)	(0.0112)	(0.0163)
30	25	16	I	μ	0.7470	0.1170	0.3326	0.1432	0.1674	0.0812
					(0.5952)	(0.0175)	(0.2722)	(0.0441)	(0.0646)	(0.0037)
				τ	0.1980	0.1552	0.1945	0.0730	0.0906	0.1661
					(0.0538)	(0.0483)	(0.0396)	(0.0144)	(0.0158)	(0.0155)
			II	μ	0.6277	0.1008	0.2710	0.1415	0.1643	0.0638
					(0.4396)	(0.0167)	(0.2059)	(0.0413)	(0.0636)	(0.0031)
				τ	0.1675	0.1520	0.1761	0.0731	0.1167	0.1545
					(0.0383)	(0.0472)	(0.0240)	(0.0119)	(0.0246)	(0.0049)
		18	I	μ	0.5822	0.0538	0.2258	0.1408	0.1664	0.0576
					(0.3814)	(0.0042)	(0.1235)	(0.0256)	(0.0337)	(0.0028)
				τ	0.1815	0.1405	0.1837	0.0712	0.0482	0.1583
					(0.0445)	(0.0284)	(0.0352)	(0.0117)	(0.0131)	(0.0130)
			II	μ	0.5082	0.0557	0.1992	0.1402	0.1658	0.0529
					(0.3019)	(0.0040)	(0.2391)	(0.0250)	(0.0333)	(0.0082)
				τ	0.1504	0.1403	0.1473	0.0709	0.0434	0.1437
					(0.0372)	(0.0306)	(0.0309)	(0.0111)	(0.0124)	(0.0099)
60	50	38	I	μ	0.7215	0.1208	0.3517	0.0958	0.1689	0.0911
					(0.5395)	(0.0184)	(0.1457)	(0.0232)	(0.0457)	(0.0419)
				τ	0.0987	0.0940	0.2446	0.0212	0.0388	0.0989
					(0.0146)	(0.0121)	(0.0643)	(0.0070)	(0.0139)	(0.0752)
			II	μ	0.7119	0.1192	0.3503	0.0903	0.1567	0.0853
					(0.5248)	0.0204	(0.1447)	(0.0145)	(0.0443)	(0.0420)
				τ	0.0901	0.0860	0.1620	0.0210	0.0382	0.1423
					(0.0221)	(0.0111)	(0.0312)	(0.0072)	(0.0133)	(0.0704)
		42	I	μ	0.6014	0.1207	0.2842	0.0891	0.1095	0.0650
					(0.3794)	(0.0196)	(0.1041)	(0.0226)	(0.0244)	(0.0371)
				τ	0.0979	0.0994	0.1992	0.0114	0.0361	0.0600
					(0.0147)	(0.0025)	(0.0444)	(0.0067)	(0.0149)	(0.0599)
			II	μ	0.5698	0.1121	0.2793	0.0857	0.0901	0.0623
					(0.3432)	(0.0278)	(0.1003)	(0.0257)	(0.0294)	(0.0291)
				τ	0.0906	0.0886	0.1072	0.0111	0.0359	0.0344
					(0.0233)	(0.0050)	(0.0176)	(0.0065)	(0.0147)	(0.0658)
60	55	38	I	μ	0.7191	0.1048	0.3474	0.0750	0.0958	0.0835
					(0.5353)	(0.0361)	(0.1427)	(0.0190)	(0.0230)	(0.0219)
				τ	0.0982	0.0892	0.2418	0.0195	0.0321	0.0929
					(0.0151)	(0.0104)	(0.0628)	(0.0067)	(0.0143)	(0.0731)
			II	μ	0.6193	0.0936	0.3391	0.0670	0.0678	0.0761
					(0.4036)	(0.0347)	(0.1357)	(0.0196)	(0.0196)	(0.0216)
				τ	0.0978	0.0854	0.1627	0.0205	0.0359	0.0284
					(0.0178)	(0.0074)	(0.0317)	(0.0068)	(0.0211)	(0.0616)
		42	I	μ	0.6007	0.0961	0.1976	0.0357	0.0839	0.0726
					(0.3786)	(0.0124)	(0.0639)	(0.0185)	(0.0217)	(0.0458)
				τ	0.0933	0.0929	0.1970	0.0107	0.0314	0.0569
					(0.0135)	(0.0013)	(0.0433)	(0.0062)	(0.0138)	(0.0594)
			II	μ	0.4617	0.1122	0.1879	0.0390	0.0820	0.0582
					(0.2329)	(0.0287)	(0.0597)	(0.0145)	(0.0215)	(0.0280)
				τ	0.0922	0.0885	0.1072	0.0195	0.0322	0.0649
					(0.0130)	(0.0019)	(0.0176)	(0.0066)	(0.0211)	(0.0142)
100	80	62	I	μ	0.6573	0.0914	0.2592	0.0942	0.1374	0.0889
					(0.4440)	(0.0158)	(0.0635)	(0.0237)	(0.0370)	(0.0128)
				τ	0.0980	0.0719	0.3620	0.1224	0.1762	0.0783
					(0.0129)	(0.0068)	(0.1515)	(0.0673)	(0.0394)	(0.0232)
			II	μ	0.5449	0.0903	0.1854	0.0933	0.1131	0.0790
					(0.3092)	(0.0158)	(0.0371)	(0.0227)	(0.0328)	(0.0198)
				τ	0.3153	0.0586	0.2404	0.1223	0.1400	0.0693
					(0.1125)	(0.0030)	(0.0718)	(0.0626)	(0.0398)	(0.0102)
		70	I	μ	0.5270	0.0606	0.2337	0.0866	0.1274	0.0680
					(0.2887)	(0.0113)	(0.0771)	(0.0172)	(0.0399)	(0.0135)
				τ	0.0766	0.0452	0.0906	0.1219	0.1624	0.0780
					(0.0089)	(0.0029)	(0.0158)	(0.0673)	(0.0396)	(0.0235)
			II	μ	0.4108	0.0604	0.1592	0.0741	0.1244	0.0675
					(0.1809)	(0.0117)	(0.0763)	(0.0160)	(0.0350)	(0.0159)
				τ	0.2102	0.0438	0.0412	0.0972	0.1377	0.0686
					(0.0540)	(0.0008)	(0.0112)	(0.0169)	(0.0369)	(0.0235)
100	90	62	I	μ	0.6531	0.0873	0.2393	0.0980	0.1303	0.0897
					(0.4416)	(0.0151)	(0.0797)	(0.0169)	(0.0359)	(0.0118)
				τ	0.0949	0.0610	0.1167	0.6563	0.1721	0.0789
					(0.0125)	(0.0029)	(0.0246)	(0.4456)	(0.0395)	(0.0227)
			II	μ	0.4123	0.0758	0.2012	0.2125	0.1255	0.0716
					(0.1837)	(0.0122)	(0.0714)	(0.1698)	(0.0360)	(0.0240)
				τ	0.1182	0.0510	0.0896	0.1402	0.1669	0.0685
					(0.0212)	(0.0030)	(0.0154)	(0.0851)	(0.0390)	(0.0212)
		70	I	μ	0.5270	0.0539	0.1512	0.0955	0.1133	0.0676
					(0.2800)	(0.0053)	(0.0880)	(0.0169)	(0.0898)	(0.0232)
				τ	0.0745	0.0488	0.0482	0.1311	0.1727	0.0728
					(0.0084)	(0.0008)	(0.0131)	(0.0766)	(0.0947)	(0.0236)
			II	μ	0.2639	0.0298	0.0921	0.0909	0.1266	0.0545
					(0.0827)	(0.0241)	(0.0299)	(0.0170)	(0.0929)	(0.0211)
				τ	0.0721	0.0421	0.0398	0.1266	0.1548	0.0669
					(0.0087)	(0.0008)	(0.0102)	(0.0668)	(0.0935)	(0.0237)

**Table A4:** ABs and MSEs (within bracket) of different estimates with μ = 0.5, τ = 1 when *T* = 0.6.

*n*	*m*	*k*	Sch	Par	SEL	LL		GEL		IS
						*ℏ* = 0.35	*ℏ* = 0.45	*q* = 0.8	*q* = 1.1	
30	20	16	I	μ	0.7811	0.1161	0.3833	0.1537	0.1738	0.0949
					(0.6541)	(0.0172)	(0.2961)	(0.0531)	(0.0731)	(0.0029)
				τ	0.1956	0.1554	0.1985	0.0740	0.0896	0.1697
					(0.0500)	(0.0323)	(0.0433)	(0.0117)	(0.0154)	(0.0182)
			II	μ	0.6270	0.1147	0.3802	0.1388	0.0896	0.0905
					(0.4396)	(0.0167)	(0.2438)	(0.0372)	(0.0154)	(0.0028)
				τ	0.1675	0.1518	0.1957	0.0653	0.0398	0.1687
					(0.0549)	(0.0321)	(0.0400)	(0.0107)	(0.0102)	(0.0164)
		18	I	μ	0.6715	0.0551	0.2316	0.1476	0.1719	0.0889
					(0.4910)	(0.0039)	(0.1054)	(0.0488)	(0.0728)	(0.0025)
				τ	0.1831	0.1403	0.1973	0.0381	0.0526	0.1573
					(0.0455)	(0.0306)	(0.0420)	(0.0091)	(0.0192)	(0.0127)
			II	μ	0.5067	0.0556	0.1959	0.1466	0.1700	0.0805
					(0.3001)	(0.0041)	(0.1296)	(0.0412)	(0.0711)	(0.0050)
				τ	0.1815	0.1402	0.1867	0.1661	0.0420	0.1547
					(0.0422)	(0.0299)	(0.0365)	(0.0381)	(0.0112)	(0.0123)
30	25	16	I	μ	0.7793	0.1895	0.2297	0.1388	0.1619	0.0629
					(0.6508)	(0.0456)	(0.1262)	(0.0372)	(0.0638)	(0.0074)
				τ	0.1975	0.1625	0.1846	0.0718	0.1194	0.1557
					(0.0508)	(0.0394)	(0.0356)	(0.0119)	(0.0263)	(0.0122)
			II	μ	0.5042	0.1608	0.1709	0.1385	0.1610	0.0605
					(0.2968)	(0.0840)	(0.1049)	(0.0372)	(0.0638)	(0.0077)
				τ	0.1442	0.5328	0.1466	0.0026	0.0911	0.1241
					(0.0365)	(0.3006)	(0.0227)	(0.0075)	(0.0158)	(0.0046)
		18	I	μ	0.6717	0.1749	0.2249	0.1167	0.1592	0.0575
					(0.4918)	(0.0328)	(0.1260)	(0.0246)	(0.0576)	(0.0159)
				τ	0.1759	0.1419	0.1937	0.0759	0.0534	0.1592
					(0.0422)	(0.0291)	(0.0392)	(0.0121)	(0.0124)	(0.0113)
			II	μ	0.4176	0.1583	0.3992	0.1097	0.1564	0.0317
					(0.2249)	(0.0426)	(0.2387)	(0.0326)	(0.0573)	(0.0373)
				τ	0.1405	0.1358	0.1419	0.0458	0.0379	0.1433
					(0.0363)	(0.0238)	(0.0262)	(0.0099)	(0.0110)	(0.0096)
60	50	38	I	μ	0.7202	0.1193	0.3516	0.0945	0.1918	0.0862
					(0.5371)	0.0203	(0.1445)	(0.0231)	(0.0979)	(0.0416)
				τ	0.0999	0.0941	0.2423	0.0238	0.0384	0.0451
					(0.0151)	(0.0124)	(0.0631)	(0.0071)	(0.0149)	(0.0689)
			II	μ	0.7103	0.1182	0.3491	0.0947	0.1605	0.8068
					(0.5229)	0.0190	(0.1432)	(0.0239)	(0.0283)	(0.0415)
				τ	0.0975	0.0851	0.0602	0.0236	0.0379	0.0612
					(0.0149)	(0.0110)	(0.0102)	(0.0070)	(0.0105)	(0.0677)
		42	I	μ	0.6029	0.1275	0.2732	0.0960	0.1700	0.0726
					(0.3818)	0.0104	(0.0975)	(0.0292)	(0.0901)	(0.1899)
				τ	0.0912	0.0987	0.2011	0.0106	0.0370	0.0588
					(0.0134)	(0.0103)	(0.0451)	(0.0065)	(0.0140)	(0.0700)
			II	μ	0.5708	0.1129	0.2700	0.0820	0.1698	0.0562
					(0.3435)	0.0274	(0.0945)	(0.0215)	(0.0900)	(0.0149)
				τ	0.0910	0.0884	0.0324	0.0250	0.0396	0.0612
					(0.0112)	(0.0267)	(0.0091)	(0.0059)	(0.0230)	(0.0834)
60	55	38	I	μ	0.7190	0.1046	0.3471	0.0773	0.1534	0.0719
					(0.5364)	0.0361	(0.1421)	(0.0249)	(0.0398)	(0.0210)
				τ	0.1002	0.0892	0.2431	0.0197	0.0320	0.0808
					(0.0149)	(0.0259)	(0.0634)	(0.0066)	(0.0143)	(0.0777)
			II	μ	0.6198	0.0935	0.3414	0.0903	0.1315	0.6755
					(0.4035)	0.0448	(0.1385)	(0.0245)	(0.0326)	(0.0203)
				τ	0.0976	0.0846	0.1618	0.0207	0.0320	0.0899
					(0.0142)	(0.0047)	(0.0383)	(0.0066)	(0.0143)	(0.0594)
		42	I	μ	0.6000	0.1260	0.1941	0.0793	0.1698	0.0713
					(0.3784)	0.0124	(0.0604)	(0.0251)	(0.0913)	(0.0478)
				τ	0.0912	0.0927	0.1942	0.0101	0.0315	0.0856
					(0.0134)	(0.0271)	(0.0430)	(0.0064)	(0.0137)	(0.0872)
			II	μ	0.4604	0.1124	0.1933	0.0529	0.0659	0.0376
					(0.2308)	0.0280	(0.0595)	(0.0222)	(0.0187)	(0.0146)
				τ	0.0911	0.0884	0.1080	0.0187	0.0529	0.1554
					(0.0129)	(0.0201)	(0.0180)	(0.0066)	(0.0215)	(0.0799)
100	80	62	I	μ	0.6559	0.0885	0.2590	0.0987	0.1269	0.0893
					(0.4429)	(0.0153)	(0.1089)	(0.0169)	(0.0993)	(0.0120)
				τ	0.0920	0.0946	0.0896	0.1354	0.1648	0.0856
					(0.0137)	(0.0062)	(0.0154)	(0.0657)	(0.0938)	(0.0209)
			II	μ	0.5476	0.0710	0.1893	0.0882	0.1227	0.0813
					(0.3123)	(0.0111)	(0.0394)	(0.0160)	(0.0915)	(0.0199)
				τ	0.3132	0.0932	0.0676	0.1232	0.1550	0.0725
					(0.1115)	(0.0042)	(0.0172)	(0.0672)	(0.0970)	(0.0215)
		70	I	μ	0.5263	0.0698	0.2399	0.0997	0.1233	0.0732
					(0.2883)	(0.0100)	(0.1005)	(0.0157)	(0.0925)	(0.0224)
				τ	0.0782	0.0590	0.0526	0.1332	0.1745	0.0780
					(0.0091)	(0.0053)	(0.0192)	(0.0660)	(0.0944)	(0.0234)
			II	μ	0.4096	0.0695	0.1228	0.0838	0.1021	0.0679
					(0.1797)	(0.0109)	(0.0373)	(0.0157)	(0.0895)	(0.0161)
				τ	0.2091	0.0371	0.0420	0.1114	0.1507	0.0690
					(0.0545)	(0.0008)	(0.0112)	(0.0683)	(0.0943)	(0.0235)
100	90	62	I	μ	0.6549	0.0543	0.2290	0.0800	0.1315	0.0892
					(0.4404)	(0.0054)	(0.0737)	(0.0150)	(0.0920)	(0.0121)
				τ	0.0977	0.0668	0.1194	0.1203	0.1690	0.0771
					(0.0130)	(0.0043)	(0.0263)	(0.0675)	(0.0952)	(0.0240)
			II	μ	0.4217	0.0423	0.2023	0.0945	0.1239	0.0720
					(0.1917)	(0.0078)	(0.0812)	(0.0164)	(0.0994)	(0.0236)
				τ	0.0732	0.0582	0.0911	0.1451	0.1567	0.0726
					(0.0087)	(0.0010)	(0.0158)	(0.0644)	(0.0968)	(0.0211)
		70	I	μ	0.5258	0.0411	0.1542	0.0845	0.1307	0.0683
					(0.2854)	(0.0075)	(0.0968)	(0.0579)	(0.0906)	(0.0237)
				τ	0.0738	0.0363	0.0534	0.1285	0.1695	0.0634
					(0.0080)	(0.0008)	(0.0124)	(0.0666)	(0.0952)	(0.0239)
			II	μ	0.2537	0.0318	0.0867	0.0794	0.1248	0.0664
					(0.0763)	(0.0004)	(0.0285)	(0.0099)	(0.0938)	(0.0239)
				τ	0.1168	0.0210	0.0379	0.1125	0.1616	0.0689
					(0.0208)	(0.0016)	(0.0110)	(0.0682)	(0.0390)	(0.0239)

**Table A5:** CPs and ALs (within bracket) of 95% CIs for the parameters with μ = 0.5, τ = 1, when *T* = 0.4.

*n*	*m*	*k*	Sch	ACIs		Log-CIs		Boot-p CIs		HPD Intervals	
				μ	τ	μ	τ	μ	τ	μ	τ
30	20	16	I	91.00%	85.32%	91.14%	91.23%	91.36%	91.40%	91.18%	91.12%
				(0.8139)	(1.4571)	(0.8044)	(1.5610)	(0.8172)	(1.3819)	(0.8034)	(1.5568)
			II	91.60%	83.21%	92.26%	92.63%	92.48%	92.18%	91.46%	91.38%
				(0.8134)	(1.3467)	(0.8634	(1.4140)	(0.8653)	(1.2618)	(0.8556)	(1.3877)
		18	I	92.60%	85.73%	90.71%	91.03%	91.82%	92.18%	91.14%	91.92%
				(0.7731)	(1.3532)	(0.7691)	(1.4545)	(0.7771)	(1.3162)	(0.7691)	(1.4527)
			II	93.40%	89.62%	94.43%	92.50%	92.64%	92.84%	91.52%	92.16%
				(0.8485)	(1.2287)	(0.8429)	(1.2999)	(0.8384)	(1.1713)	(0.8407)	(1.2888)
30	25	16	I	92.70%	85.10%	91.82%	91.92%	92.03%	91.70%	91.72%	91.60%
				(0.8212)	(1.3716)	(0.8065)	(1.5668)	(0.8231)	(1.4005)	(0.7712)	(1.4605)
			II	92.20%	86.23%	92.93%	93.32%	92.48%	92.02%	91.84%	91.88%
				(0.8172)	(1.3527)	(0.8198)	(1.4811)	(0.8272)	(1.3351)	(0.7948)	(1.3854)
		18	I	92.30%	84.55%	93.56%	92.30%	91.78%	92.35%	91.68%	92.22%
				(0.7684)	(1.3399)	(0.7656)	(1.4376)	(0.7724)	(1.3021)	(0.7681)	(1.4486)
			II	0.929	85.76%	92.18%	91.70%	92.32%	92.38%	91.68%	92.52%
				(0.7910)	(1.2766)	(0.7928)	(1.3786)	(0.7897)	(1.2489)	(0.7920)	(1.3751)
60	50	38	I	92.57%	88.64%	93.07%	93.62%	93.20%	91.54%	93.50%	93.80%
				0.5479)	(0.9431)	(0.5459)	(0.9824)	(0.5368)	(0.9170)	(0.5158)	(0.7909)
			II	93.21%	89.90%	94.16%	93.74%	93.70%	92.80%	93.62%	93.82%
				(0.5183)	(0.8916)	(0.5656)	(0.9396)	(0.5316)	(0.8707)	(0.5085)	(0.7485)
		42	I	93.53%	90.21%	94.08%	94.03%	96.24%	92.50%	93.78%	94.08%
				(0.5436)	(0.9493)	(0.5270)	(0.9147)	(0.5412)	(0.8654)	(0.5289)	(0.7429)
			II	93.92%	91.37%	92.19%	93.62%	93.81%	92.67%	93.91%	94.16%
				(0.5090)	(0.9156)	(0.5517)	(0.8698)	(0.5194)	(0.8479)	(0.5153)	(0.7259)
60	55	38	I	93.27%	90.23%	94.12%	93.33%	93.50%	92.20%	93.67%	94.48%
				(0.5312)	(0.8481)	(0.5439)	(0.9763)	(0.5431)	(0.9157)	(0.4972)	(0.7352)
			II	93.64%	91.68%	93.71%	94.31%	93.60%	93.30%	93.82%	94.36%
				(0.5292)	(0.8462)	(0.5559)	(0.9686)	(0.5345)	(0.8917)	(0.5019)	(0.7353)
		42	I	93.66%	90.51%	94.13%	93.86%	93.91%	92.91%	94.16%	94.50%
				(0.5150)	(0.9174)	(0.5323)	(0.9334)	(0.5273)	(0.8644)	(0.5066)	(0.7076)
			II	93.91%	91.03%	94.35%	94.51%	94.15%	93.73%	94.40%	94.93%
				(0.5452)	(0.7920)	(0.5421)	(0.9092)	(0.5175)	(0.8249)	(0.4912)	(0.7031)
100	80	62	I	93.25%	90.28%	94.80%	93.90%	93.52%	90.50%	94.86%	95.08%
				(0.4313)	(0.8709)	(0.4294)	(0.7688)	(0.4361)	(0.7311)	(0.4299)	(0.6750)
			II	94.10%	91.56%	93.21%	94.03%	94.23%	91.57%	94.38%	95.60%
				(0.4386)	(0.7537)	(0.4476)	(0.7270)	(0.4172)	(0.6800)	(0.4148)	(0.6714)
		70	I	93.71%	91.74%	95.26%	93.24%	93.73%	92.03%	95.72%	95.24%
				(0.4129)	(0.6561)	(0.4131)	(0.7126)	(0.4219)	(0.6807)	(0.4138)	(0.6603)
			II	94.82%	92.36%	93.33%	92.53%	94.96%	92.40%	94.34%	95.52%
				(0.4228)	(0.6480)	(0.4354)	(0.6686)	(0.3985)	(0.6311)	(0.4180)	(0.6478)
100	90	62	I	94.5%	91.93%	95.28%	95.30%	94.56%	92.59%	95.74%	95.64%
				(0.4314)	(0.8907)	(0.4327)	(0.7778)	(0.4234)	(0.7243)	(0.4017)	(0.6513)
			II	94.64%	92.03%	94.62%	94.06%	94.50%	92.50%	94.88%	96.02%
				(0.4311)	(0.9071)	(0.4387)	(0.7569)	(0.4209)	(0.7078)	(0.3929)	(0.6138)
		70	I	94.71%	91.92%	95.17%	95.10%	94.88%	92.03%	95.03%	96.04%
				(0.4380)	(0.7111)	(0.4152)	(0.7195)	(0.4198)	(0.6256)	(0.3987)	(0.6131)
			II	94.83%	92.65%	95.63%	93.70%	95.01%	92.80%	95.97%	96.78%
				(0.4134)	(0.6985)	(0.4242)	(0.6977)	(0.3867)	(0.5862)	(0.3716)	(0.5252)

**Table A6:** CPs and ALs (within bracket) of 95% CIs for the parameters with μ = 0.5, τ = 1, when *T* = 0.6.

*n*	*m*	*k*	Sch	ACIs		Log-CIs		Boot-p CIs		HPD Intervals	
				μ	τ	μ	τ	μ	τ	μ	τ
30	20	16	I	92.21%	85.12%	92.80%	91.37%	91.86%	91.54%	91.28%	91.36%
				(0.8082)	(1.4333)	(0.8086)	(1.5806)	(0.8245)	(1.3886)	(0.8094)	(1.3806)
			II	92.63%	85.12%	93.54%	91.92%	93.20%	92.68%	91.10%	92.63%
				(0.8552)	(1.2903)	(0.8567)	(1.3925)	(0.8575)	(1.2579)	(0.8610)	(1.2051)
		18	I	92.42%	83.91%	92.73%	91.11%	90.90%	92.36%	91.33%	91.64%
				(0.7682)	(1.3373)	(0.7644)	(1.4355)	(0.7752)	(1.311)	(0.7709)	(1.2593)
			II	91.70%	87.90%	92.31%	92.00%	92.52%	92.94%	91.42%	92.78%
				(0.8416)	(1.2125)	(0.8379)	(1.2806)	(0.8335)	(1.1767)	(0.8422)	(1.1927)
30	25	16	I	91.66%	85.00%	91.93%	92.73%	91.42%	91.58%	91.34%	91.85%
				(0.8136)	(1.4535)	(0.8081)	(1.5726)	(0.8180)	(1.3935)	(0.8094)	(1.3579)
			II	92.81%	86.08%	91.00%	92.48%	91.98%	92.26%	92.52%	91.36%
				(0.856)	(1.2924)	(0.8239)	(1.4979)	(0.8218)	(1.3367)	(0.8240)	(1.2990)
		18	I	91.80%	85.53%	92.71%	91.40%	91.62%	91.48%	92.14%	92.14%
				(0.7705)	(1.3431)	(0.7663)	(1.4428)	(0.7846)	(1.3042)	(0.7673)	(1.2944)
			II	92.93%	86.32%	92.80%	91.73%	92.06%	92.00%	92.46%	92.38%
				(0.7971)	(1.2958)	(0.7905)	(1.3683)	(0.7961)	(1.2453)	(0.7911)	(1.2373)
60	50	38	I	93.33%	89.20%	95.40%	94.60%	93.40%	94.53%	93.46%	94.76%
				(0.5389)	(0.9433)	(0.5480)	(0.9901)	(0.5554)	(0.9074)	(0.5162)	(0.7907)
			II	93.58%	88.87%	93.52%	93.33%	93.80%	94.10%	94.00%	94.20%
				(0.5491)	(0.9340)	(0.5672)	(0.9454)	(0.5378)	(0.8759)	(0.5066)	(0.7491)
		42	I	93.74%	90.10%	93.21%	94.61%	93.85%	94.33%	94.10%	94.84%
				(0.4133)	(0.8990)	(0.5286)	(0.9198)	(0.5426)	(0.8560)	(0.5290)	(0.7463)
			II	94.00%	91.34%	95.73%	93.82%	94.00%	94.50%	94.20%	94.62%
				(0.5338)	(0.9572)	(0.5551)	(0.8818)	(0.5124)	(0.8221)	(0.5143)	(0.7439)
60	55	38	I	94.31%	90.62%	93.20%	92.79%	94.50%	94.87%	94.40%	95.10%
				(0.4308)	(0.8645)	(0.5422)	(0.9686)	(0.5301)	(0.8556)	(0.5077)	(0.7355)
			II	94.22%	90.62%	93.10%	93.64%	94.31%	94.80%	94.55%	95.50%
				(0.4474)	(0.8358)	(0.5524)	(0.9559)	(0.5084)	(0.8406)	(0.4970)	(0.7103)
		42	I	93.50%	90.71%	93.66%	94.00%	93.58%	94.93%	94.42%	95.78%
				(0.5529)	(0.8817)	0.5572)	(0.9157)	(0.5263)	(0.8129)	(0.5031)	(0.7025)
			II	94.92%	91.63%	93.00%	94.44%	94.81%	94.90%	94.70%	96.08%
				(0.4365)	(0.8042)	(0.5417)	(0.9079)	(0.5210)	(0.7729)	(0.4963)	(0.6911)
100	80	62	I	93.70%	90.91%	93.91%	93.85%	93.70%	91.00%	94.00%	95.20%
				(0.4319)	(0.7260)	(0.4278)	(0.7610)	(0.4374)	(0.6958)	(0.4206)	(0.6753)
			II	93.81%	92.00%	93.90%	95.43%	94.00%	92.50%	94.06%	95.66%
				(0.4390)	(0.7574)	(0.4509)	(0.7375)	(0.4125)	(0.6858)	(0.4112)	(0.6711)
		70	I	93.76%	91.63%	94.70%	95.20%	94.12%	92.80%	94.54%	95.22%
				(0.4125)	(0.6491)	(0.4128)	(0.0717)	(0.4191)	(0.6545)	(0.4038)	(0.6472)
			II	94.06%	92.22%	94.50%	94.36%	94.31%	92.28%	94.68%	95.10%
				(0.4240)	(0.6150)	(0.4379)	(0.6721)	(0.4120)	(0.6355)	(0.4022)	(0.6312)
100	90	62	I	94.45%	92.10%	94.00%	93.57%	94.20%	93.07%	94.66%	96.40%
				(0.4011)	(0.7081)	(0.4312)	(0.7731)	(0.4243)	(0.7031)	(0.4018)	(0.6516)
			II	94.70%	92.70%	94.03%	94.51%	94.85%	93.07%	94.90%	96.28%
				(0.4380)	(0.6986)	(0.4377)	(0.7531)	(0.4325)	(0.6733)	(0.3921)	(0.6402)
		70	I	95.31%	92.16%	93.76%	94.11%	95.55%	92.90%	95.81%	96.38%
				(0.4008)	(0.7392)	(0.4142)	(0.7164)	(0.3951)	(0.6256)	(0.3925)	(0.6113)
			II	95.50%	93.95%	94.81%	93.90%	95.89%	94.10%	96.01%	96.14%
				(0.4313)	(0.6955)	(0.4236)	(0.6954)	(0.3893)	(0.6090)	(0.3756)	(0.5907)
